# Supplementary material for: Regulation of NGF Signaling by an Axonal Untranslated mRNA
Source: Neuron. 2019 May 8;102(3):553–563.e8. doi: 10.1016/j.neuron.2019.02.011 (PMC6509357; doi:10.1016/j.neuron.2019.02.011)
Supplement: Document S1. Figures S1–S4 and Tables S1 and S2 [file mmc1.pdf]

Neuron, Volume *102*

Supplemental Information

Regulation of NGF Signaling

by an Axonal Untranslated mRNA

Hamish Crerar, Emily Scott-Solomon, Chantal Bodkin-Clarke, Catia Andreassi, Maria Hazbon, Emilie Logie, Marifé Cano-Jaimez, Marco Gaspari, Rejji Kuruvilla, and Antonella Riccio

Figure S1

A

GeneID	GeneSymbol	**Axon** Log2 Read Count (Average)	**Cell Body** Log2 Read Count (Average)	Axon:Cell Body Log2 Ratio
ENRNOT00000055310	Tp53inp2	18.150	9.091	1.997
ENRNOT00000015034	Trak2-202	15.205	6.627	2.294
ENRNOT00000014939	Trak2-201	15.204	6.627	2.294
ENRNOT00000030919	Fth1	14.888	9.292	1.602
ENRNOT00000071069	Nefm	13.547	12.422	1.091
ENRNOT00000027691	Apc	13.338	7.882	1.692
ENRNOT00000001383	Tpt1	13.296	8.176	1.626
ENRNOT00000004921	Sytl4-202	13.292	5.383	2.469
ENRNOT00000004909	Sytl4-201	13.292	5.382	2.470
ENRNOT00000028315	Ftl1	13.276	8.248	1.610

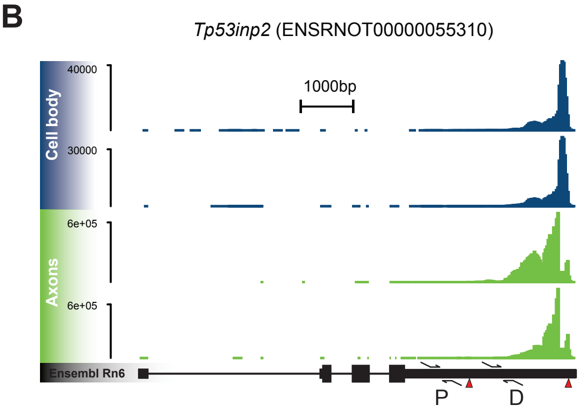

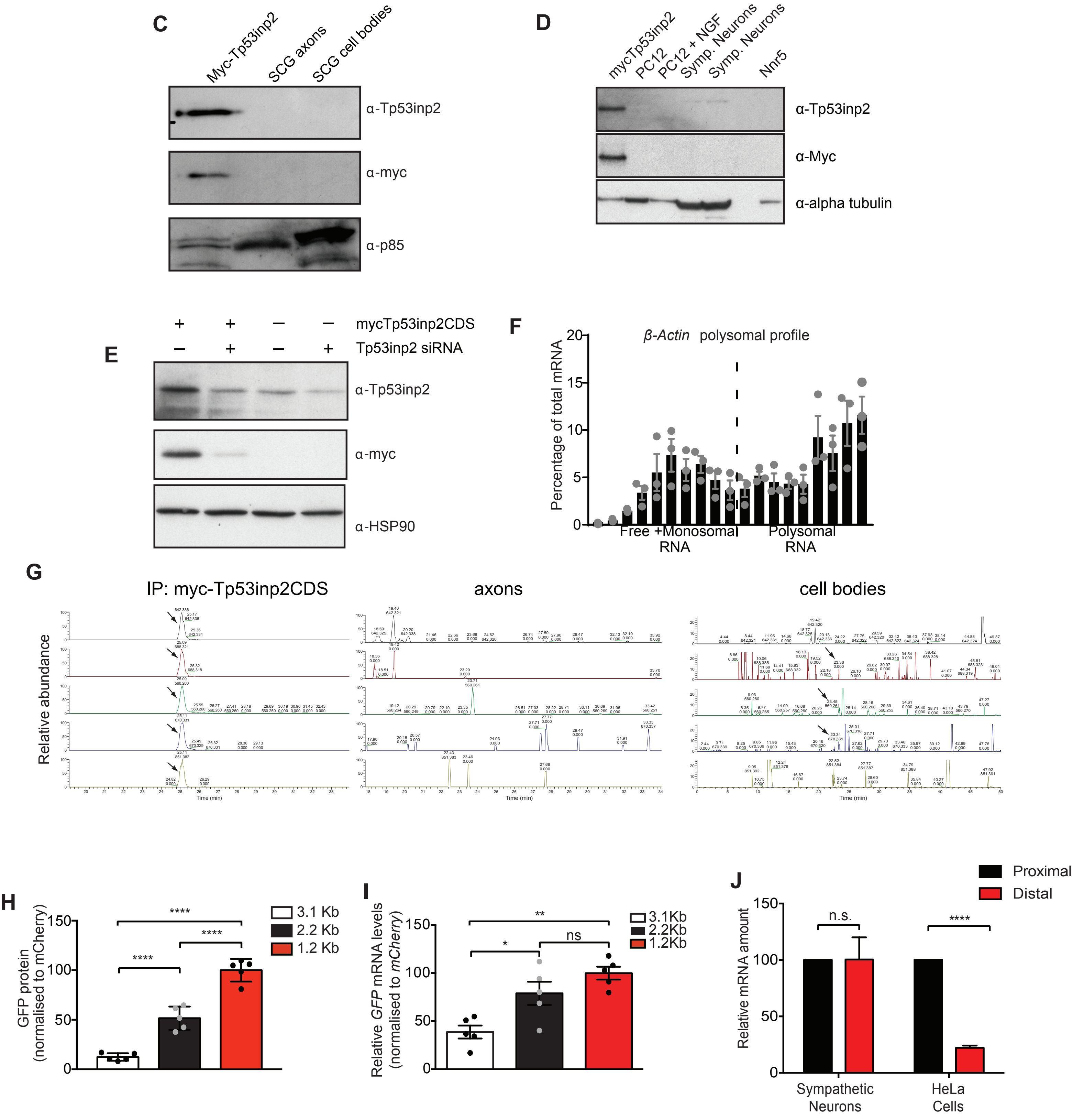

Figure S1: *Tp53inp2* translation is repressed in sympathetic neurons. Related to Figure 1. A, Top ten most abundant axonal transcripts as identified by 3'end RNAseq screen performed on axons and cell bodies of sympathetic neurons. **B**, (*upper*) 3'end Seq tracks of *Tp53inp2* mRNA from cell body and axonal mRNA and (l*ower*) schematic of Ensembl transcript ID ENSRNOT00000055310, red arrowhead represent the positions of AATAAA PAS hexamer, black arrows represent the position of proximal (P) and distal (D) primers used for analysis in **Figure S1J**. **C**, Western blotting analysis of Tp53inp2, Myc and PI3K subunit of p85 on PC12 lysates transfected with Myc-Tp53inp2 CDS, and axonal and cell body lysates of sympathetic neurons. See also (**Figure 1A,B**). **D**, Western blot analysis of PC12 cells transfected with mycTp53inp2, PC12 cells ±NGF stimulation, SCG sympathetic neurons and Nnr5 PC12 cell line. **E**, Western blot analysis of HeLa lysates co-transfected with mycTp53inp2CDS and Tp53inp2 siRNA, as indicated (*n*=3). (See also **Figure 1B**). **F,** Polysomal fractionation profile of *β-actin* mRNA from sympathetic neurons. Values are expressed as percentage of total *β-actin* mRNA (*n*=3). (See also **Figure 1C**). **G,** *Pseudo*-Selected Reaction Monitoring traces for the detection of a Tp53inp2 tryptic peptide in cultured sympathetic neuron axon or cell body samples, and in immunoprecipitated mycTp53inp2 control. The 5 traces represent the 5 most abundant fragments of the Tp53inp2 peptide HQGSFIYQPCQR (*m/z* 507.6). Arrows indicate where at least three transitions are detected at the same retention time. Top value on trace=retention value, bottom value=m/z. (See also **Figure 1D-F**). **H**, Densitometry of GFP protein levels normalised by mCherry levels and expressed as percentage of the mean GFP protein amount of the 1.2 Kb construct. Data presented as average ±s.e.m.; Ordinary one-way ANOVA Tukey's multiple comparisons (*n*=5, ****P<0.0001). See also (**Figure 1G**). **I**, Relative *GFP* mRNA expression in PC12 cells transfected with GFP constructs carrying *Tp53inp2* 3'UTR of indicated length (Kb). mRNA levels were normalised to levels of *mCherry* mRNA and expressed as percentage of the mean *GFP* amount of the 1.2 Kb construct. Data are presented as

averages ±s.e.m., ordinary one-way Tukey's multiple comparisons ANOVA (*n*=5, *P<0.05 **P<0.01 n.s. = not statistically significant). (See also **Figure 1G**). **J,** RT-qPCR analysis of *Tp53inp2* mRNA expression in rat sympathetic neurons and HeLa cell lysate. Primers proximal and distal of the internal polyA site in rat *Tp53inp2* 3'UTR (see **Figure S1B**) were used for analysis and values are normalised to percentage expression of the respective proximal primer set. Data are presented as average ±s.e.m. multiple t-test (*n*=6, ****P<0.0001 n.s. = not statistically significant).

Figure S2

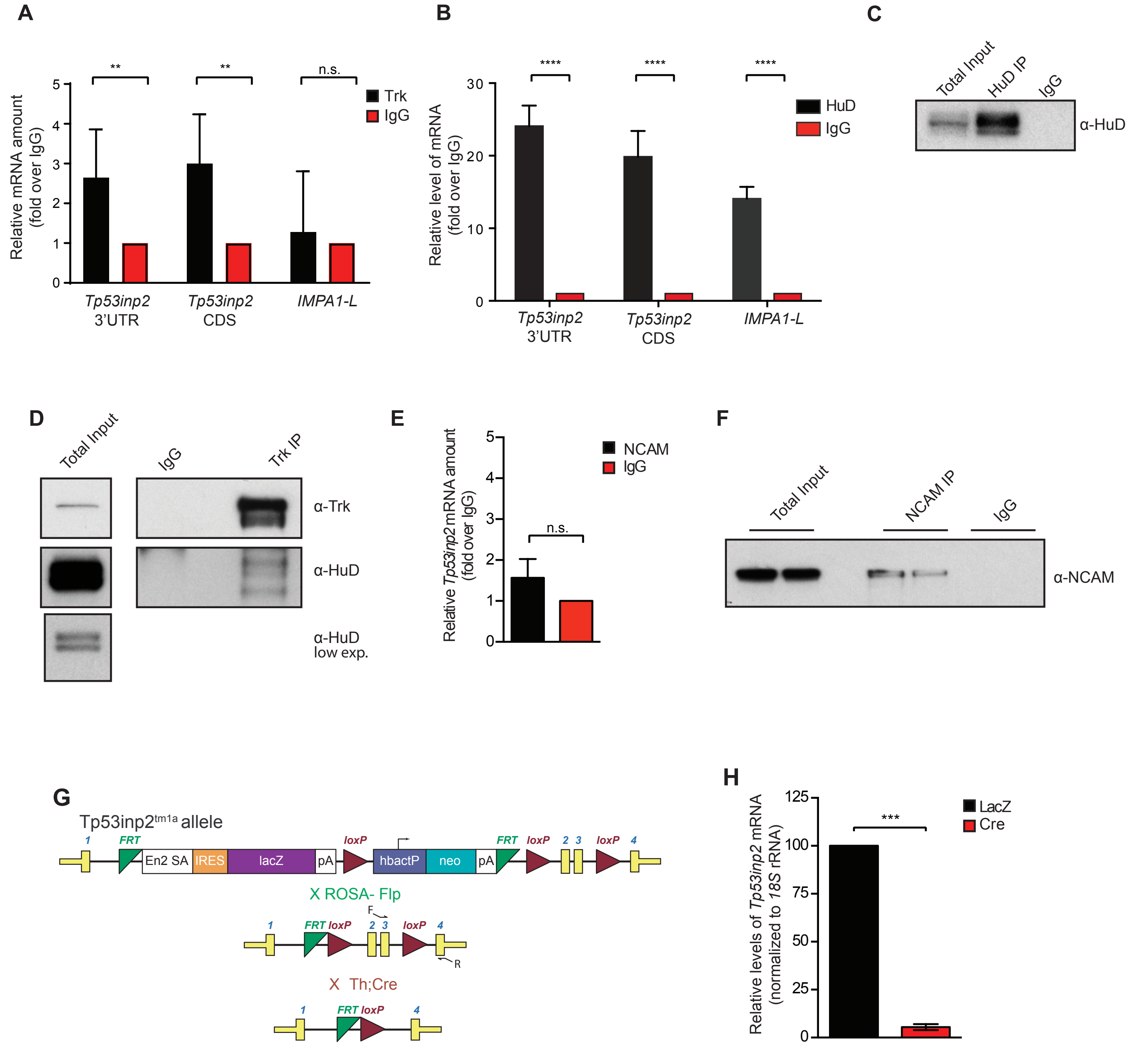
A
Relative mRNA amount (fold over IgG)
**
**
n.s.
Trk
IgG
Tp53inp2 3'UTR
Tp53inp2 CDS
IMPA1-L
B
Relative level of mRNA (fold over IgG)
****
****
****
HuD
IgG
Tp53inp2 3'UTR
Tp53inp2 CDS
IMPA1-L
C
Total Input
HuD IP
IgG
α-HuD
D
Total Input
IgG
Trk IP
α-Trk
α-HuD
α-HuD low exp.
E
Relative Tp53inp2 mRNA amount (fold over IgG)
n.s.
NCAM
IgG
F
Total Input
NCAM IP
IgG
α-NCAM
G
Tp53inp2tm1a allele
FRT
loxP
En2 SA
IRES
lacZ
pA
hbactP
neo
X ROSA- Flp
X Th;Cre
H
Relative levels of Tp53inp2 mRNA (normalized to 18S rRNA)
***
LacZ
Cre

Figure S2: *Tp53inp2* mRNA interacts with the TrkA complex to regulate its function. Related to Figure 2. A, RNA immunoprecipitation performed on sympathetic neuron lysates using antibodies for pan-Trk, or normal mouse IgG. Levels of *Tp53inp2* were analysed by RT-qPCR, normalised as fraction of total input and expressed as fold over IgG. Data are presented as average ±s.e.m. multiple t-test (*n*=4, **P<0.01 n.s. = not statistically significant). (See also **Figure 2A**). **B**, RNA immunoprecipitation performed on sympathetic neuron lysates using antibodies for HuD or normal mouse IgG. Levels of *Tp53inp2 or Impa-L* mRNAs were analysed by RT-qPCR, normalised as fraction of total input and expressed as fold of control IgG. Data are presented as average ±s.e.m. multiple t-test (*n*=4, ****P<0.0001). **C,** Western blot analysis of HuD immunoprecipitation in sympathetic neurons. **D**, Western blot analysis of Trk co-immunoprecipitation experiments in PC12 cell lysates. HuD co-immuno-precipitates with Trk. Images separated by vertical lines are taken from the same exposure. Low exposure of HuD in input sample shows presence of duplet (*n*=3). **E,** RNA immunoprecipitation performed on sympathetic neuron lysates using antibodies for NCAM or normal mouse IgG. Levels of *Tp53inp2* were analysed by RT-qPCR and expressed as fold over IgG. Data are presented as average ±s.e.m. Unpaired two tailed *T*-test with Welch's correction (*n*=4, n.s. = not statistically significant). **F**, Western blot analysis of NCAM immunoprecipitation in two representative sympathetic neuron lysates. **G**, Schematic for the generation of the transgenic *Tp53inp2*$^{fl/fl}$ and *Th-Cre;Tp53inp2*$^{fl/fl}$ mice. *Tp53inp2*^{tm1a} mice were first crossed with the ubiquitously-expressing Flippase 129S4/SvJaeSor-*Gt(ROSA)26Sortm1(FLP1)Dym/J* to excise the LacZ/neo cassette, and then with *Th-Cre* transgenic mice to generate mice lacking *Tp53inp2* in sympathetic neurons (*Th-Cre;Tp53inp2*$^{fl/fl}$ mice). Exons are indicated by yellow bars, with UTRs as projecting thin yellow bars. Location of primers used for genotyping indicated as F and R labelled black arrows. **H**, *Tp53inp2* mRNA levels in axons of *Tp53inp2*$^{fl/fl}$ SCG explants infected with adenovirus expressing LacZ or Cre. Values are normalised by

levels of *18S* rRNA and expressed as percentage of LacZ infected neurons values. Data are presented as average ±s.e.m. one-sample t-test (*n*=3 ***P<0.001). (See also **Figure 2** and **Figure 3**).

Figure S3

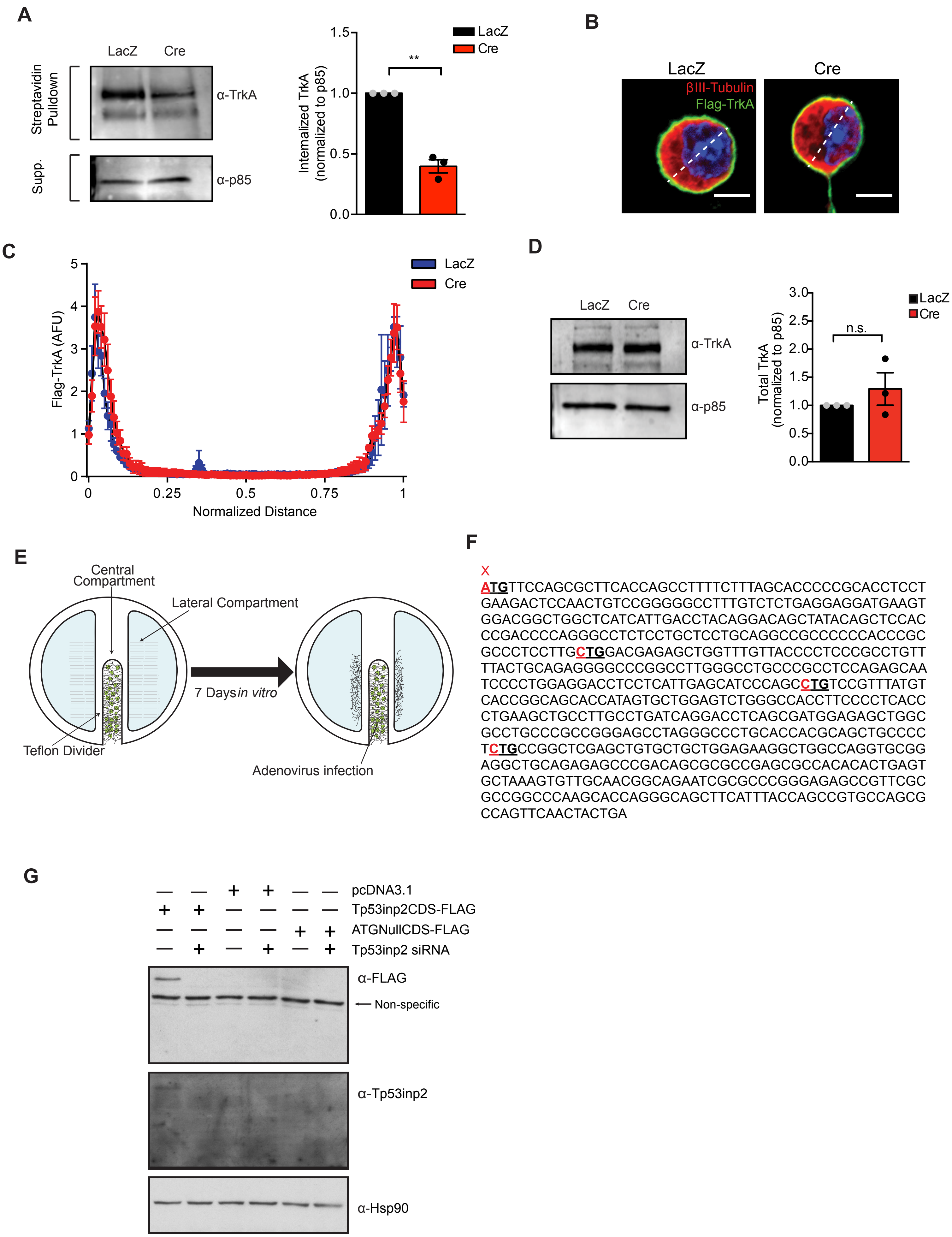
A
LacZ
Cre
Streptavidin Pulldown
Supp.
α-TrkA
α-p85
Internalized TrkA (normalized to p85)
1.5
1.0
0.5
0.0
**
LacZ
Cre
B
LacZ
Cre
βIII-Tubulin
Flag-TrkA
C
Flag-TrkA (AFU)
Normalized Distance
0
0.25
0.5
0.75
1
5
4
3
2
1
0
LacZ
Cre
D
LacZ
Cre
α-TrkA
α-p85
Total TrkA (normalized to p85)
3.0
2.5
2.0
1.5
1.0
0.5
0.0
n.s.
LacZ
Cre
E
Central Compartment
Lateral Compartment
Teflon Divider
7 Days *in vitro*
Adenovirus infection
F
X
ATGTTCCAGCGCTTCACCAGCCTTTTCTTTAGCACCCCCGCACCTCCT
GAAGACTCCAACTGTCCGGGGGCCTTTGTCTCTGAGGAGGATGAAGT
GGACGGCTGGCTCATCATTGACCTACAGGACAGCTATACAGCTCCAC
CCGACCCCAGGGCCTCTCCTGCTCCTGCAGGCCGCCCCCCACCCGC
GCCCTCCTTGCTGGACGAGAGCTGGTTTGTTACCCCTCCCGCCTGTT
TTACTGCAGAGGGGCCCGGCCTTGGGCCTGCCCGCCTCCAGAGCAA
TCCCCTGGAGGACCTCCTCATTGAGCATCCCAGCCTGTCCGTTTATGT
CACCGGCAGCACCATAGTGCTGGAGTCTGGGCCACCTTCCCCTCACC
CTGAAGCTGCCTTGCCTGATCAGGACCTCAGCGATGGAGAGCTGGC
GCCTGCCCGCCGGGAGCCTAGGGCCCTGCACCACGCAGCTGCCCC
TCTGCCGGCTCGAGCTGTGCTGCTGGAGAAGGCTGGCCAGGTGCGG
AGGCTGCAGAGAGCCCGACAGCGCGCCGAGCGCCACACACTGAGT
GCTAAAGTGTTGCAACGGCAGAATCGCGCCCGGGAGAGCCGTTCGC
GCCGGCCCAAGCACCAGGGCAGCTTCATTTACCAGCCGTGCCAGCG
CCAGTTCAACTACTGA
G
pcDNA3.1
Tp53inp2CDS-FLAG
ATGNullCDS-FLAG
Tp53inp2 siRNA
α-FLAG
Non-specific
α-Tp53inp2
α-Hsp90

Figure S3: *Tp53inp2* function in axon growth is translation independent. Related to Figure 2 and 3. A, Membrane proteins in mass cultures of *Tp53inp2*$^{fl/fl}$ neurons, infected with either LacZ or Cre, were subjected to cell surface biotinylation. After NGF stimulation for 30 minutes, surface biotin was stripped off, and internalized TrkA receptors were detected by neutravidin precipitation and TrkA immunoblotting (*Left*). Supernatants were probed for p85 as a loading control. Densitometry analysis (*Right*) of internalized biotinylated TrkA levels, normalised by p85. Values expressed as relative to LacZ-expressing neurons. Data presented as average ± s.e.m., unpaired two-tailed *t*-test (**P<0.01 *n*=3). See also (**Figure 2F, G**). **B**, Representative images of surface Flag-TrkA immunostaining in *Tp53inp2*$^{fl/fl}$ sympathetic neurons infected with adenovirus expressing Cre or LacZ and co-infected with adenovirus expressing Flag-TrkA. Neurons were live-labelled with Flag antibody in the absence of NGF at 4°C. **C**, Surface Flag-TrkA receptor distribution in *Tp53inp2*$^{fl/fl}$ sympathetic neurons infected with adenovirus expressing LacZ or Cre was analysed by measuring the integrated fluorescence values along the longest axis of the cell body using line-plot in ImageJ and normalized to the total cell body fluorescence intensity. Data are presented as averages ± s.e.m. Mann-Whitney two-tailed test (P=0.1089, *n*=3 with at least 15-20 cells analysed per condition per experiment). **D**, Western blot analysis (*Left*) of endogenous TrkA expression in *Tp53inp2*$^{fl/fl}$ neurons infected with LacZ and Cre adenoviruses, with p85 as a loading control. Densitometry analyses (*Right*) of TrkA protein levels normalized to p85. Data presented as average ± s.e.m. Unpaired two-tailed t-test (n.s.= not significant, *n*=3). **E**, Schematic representation of compartmentalised chambers. Neurons are plated in central compartment and axons project into the lateral compartments. For adenoviral infections, adenovirus is supplied to the central compartment after axons start projecting into the lateral compartments. See also (**Figure 2 and 3**). **F**, Nucleotide sequence of ATGNull CDS mutant sequence of *Tp53inp2* rat mRNA. Mutations are indicated by red underlined characters; deletion of adenosine from initial translational start codon and

substitution of adenosine to cytosine in subsequent in-frame ATG codons. See also (**Figure 3C, D**). **G,** Western blotting on lysates of PC12 cells transfected with Tp53inp2CDS-1xFlag, ATGNullCDS-1xFlag or pcDNA3.1 as control. Cells were also transfected with siRNA for *Tp53inp2* mRNA or non-targeting control siRNA (*n*=3). See also (**Figure 3C, D**).

Figure S4

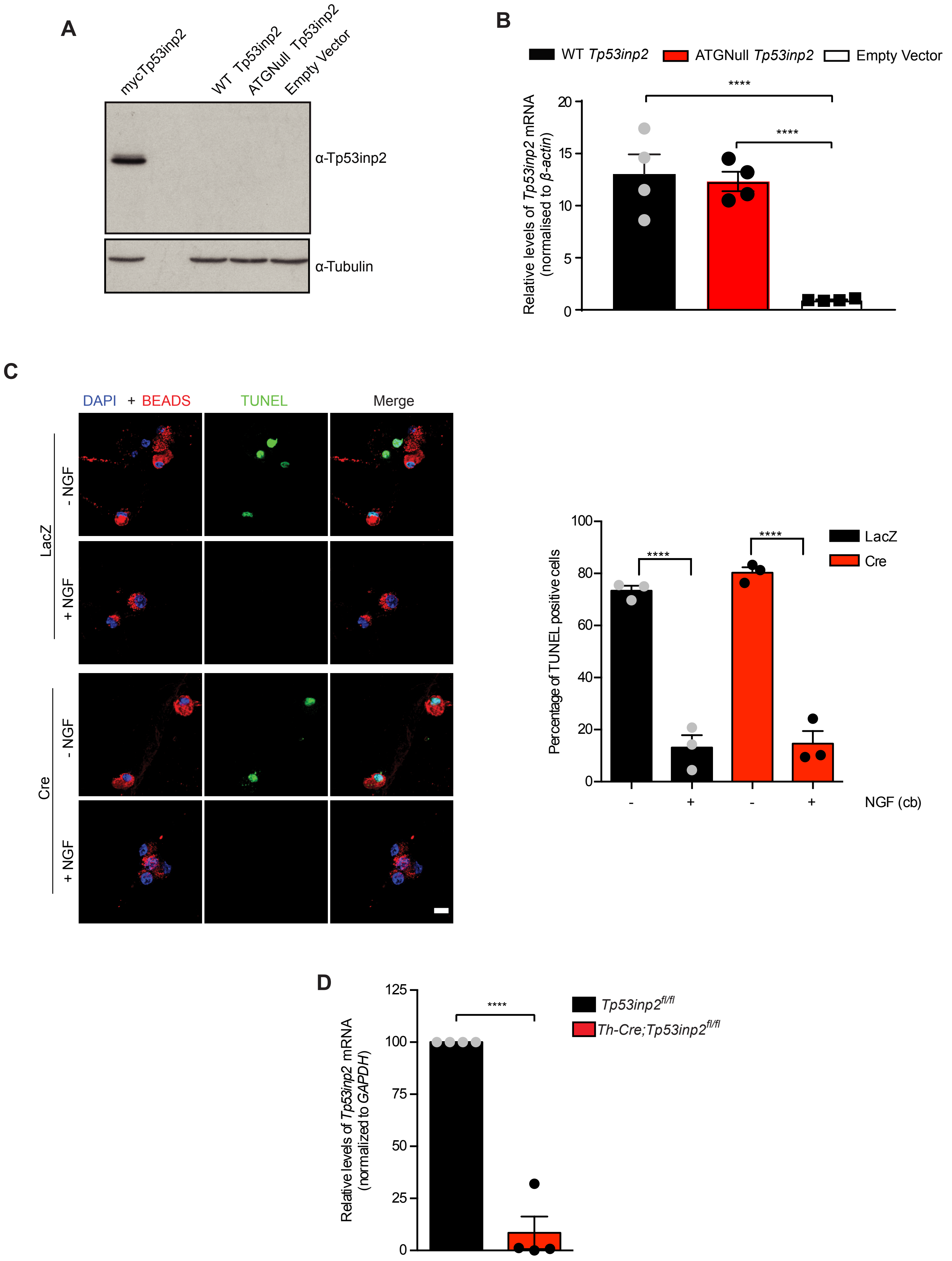
A
mycTp53inp2
WT Tp53inp2
ATGNull Tp53inp2
Empty Vector
α-Tp53inp2
α-Tubulin
B
WT Tp53inp2
ATGNull Tp53inp2
Empty Vector
Relative levels of Tp53inp2 mRNA (normalised to β-actin)
****
****
C
DAPI + BEADS
TUNEL
Merge
LacZ
- NGF
+ NGF
Cre
- NGF
+ NGF
Percentage of TUNEL positive cells
LacZ
Cre
****
****
NGF (cb)
D
Relative levels of Tp53inp2 mRNA (normalized to GAPDH)
Tp53inp2fl/fl
Th-Cre;Tp53inp2fl/fl
****

Figure S4: *Tp53inp2* is essential for the growth of NGF-responsive sympathetic neurons. Related to Figure 3 and 4. A, Western blotting analysis of PC12 cell lysates transfected with vectors expressing mycTp53inp2CDS, WildType *Tp53inp2* mRNA, full length ATGNull *Tp53inp2* or empty vector. (*n*=3). See also (**Figure 3C, D**). **B**, Expression levels of *Tp53inp2* mRNA in PC12 cells transfected with vectors expressing full length WildType *Tp53inp2* mRNA, full length ATGNull *Tp53inp2* or empty vector, as a control. Levels of *Tp53inp2* mRNA were analysed by RT-qPCR, normalised to levels of *β-actin* mRNA and expressed as fold over control *Tp53inp2* mRNA levels. Data are presented as average ±s.e.m. Ordinary one-way ANOVA (*n*=4,****P<0.0001). (See **Figure 3C, D**). **C,** *(Left)* Cell bodies of compartmentalised cultures of *Tp53inp2*$^{fl/fl}$ neurons infected with adenovirus expressing LacZ or Cre were treated with NGF (25 ng/ml) or anti-NGF antibody for 48 hours. Neurons that had projected to axonal chambers were identified through the uptake of fluorescent beads (red) supplied to the axon chambers. Neuronal apoptosis was detected using TUNEL staining. Scale bar 5 µm. *(Right)* Quantification of neuronal cell death in *Tp53inp2*$^{fl/fl}$ sympathetic neurons expressing Cre or LacZ and NGF supplied to the cell bodies (cb)**.** Data are presented as averages ± s.e.m Ordinary two-way ANOVA Tukey's multiple comparisons, (*n*=3, each data point represents the average of at least 30 cells per condition per experiment, ****P<0.0001). See also (**Figure 3E**). **D**, SCGs were harvested from P0.5 *Th-Cre;Tp53inp2*$^{fl/fl}$ mice and *Tp53inp2*$^{fl/fl}$ littermates. Levels of *Tp53inp2* mRNA were analysed by RT-qPCR, normalised by GAPDH and calculated as percentage of the *Tp53inp2*$^{fl/fl}$ values. Data are presented as average ±s.e.m. unpaired two-tailed t-test *(n*=4 mice per genotype ****P<0.0001). (See also **Figure 4**).

Table S1: List of Tp53inp2 proteotypic peptides identified in Tp53inp2 immuno-precipitate. Related to Figure 1

Peptide sequence	*m/z*	Mass error (ppm)	Charge	Retention time (min)	Ion score (Xcorr)	Main fragment ions (*m/z*)
**ALHHAAAPMoxPAR**	420.2205	-0.3	3	7.3	3.67	343.208 (y3), 530.283 (b5), 601.320 (b6), 672.357 (b7)
**HQGSFIYQPCQR**	507.5734	-0.4	3	25.1	3.58	642.336 (a6), 688.321 (y5), 560.260 (y4), 670.331 (b6), 851.381 (y6)
**ALHHAAAPMPAR**	414.8897	+1.6	3	10.3	4.97	343.208 (y3), 530.283 (b5), 601.320 (b6), 672.357 (b7), 571.302 (y5), 474.249 (y4)

Tp53inp2 tryptic peptides identified by data-dependent nanoLC-MS/MS analysis on a Tp53inp2 immunoprecipitate. Experimental *m/z*, mass error, chromatographic retention time, peptide score according to the Sequest search engine and suitable fragments for pseudo-SRM targeted analysis (based on both ion abundance and specificity) are reported.

Table S2: List of primers used in this study. Related to STAR Methods

Application	Primer Name	Primer Sequences
Genotyping	Tp53inp2LoxP3F	GATCAGGACCTCAGCGATGG
Genotyping	Tp53inp2LoxP3R	GCACCTGGCACAGGTAACTA
Cloning	NotI3UTRTpF	AAT ATA GCG GCC GCG CGC CCC CGC TG
Cloning	XhoI3UTRTp1.2R	TGC TTA CTC GAG CTC TAC CCA GGA GTG TTT ATT CC
Cloning	XhoI3UTRTp2.2R	TGC TTA CTC GAG GCA GGA GAC TGA GTA GTG TTG G
Cloning	XhoI3UTRTp3.1R	CTG GCT CGA GTT AAT GTG CTT TAA ATA GAT TTT TAT TTG
Cloning	NheI5UTRTpF	TAA GCA GCT AGC ACA ATC AGC TGT TGA AGC G
Cloning	BamHI5UTRTpR	TCT TAG GAT CCG GTC AGC GGG TGA AGC
Cloning	BamHINullCDSF	TGA GGA TCC TGT TCC AGC GCT TCA CC
Cloning	NotITpCDSR	TTC GGC GGC CGC TCA GTA GTT GAA CTG GCG
Cloning	KpnIRescueF	ACA GGT ACC ACA ATC AGC TGT TGA AGC
Cloning	EcoRVRescueR	GAC GAT ATC TGT GCT TTA AAT AGA T
Cloning	BamHINullCDSF	TCA GGA TCC TGT TCC AGC GCT TC
Cloning	BamHITpCDSF	TTC AGG ATC CAT GTT CCA GCG CTT C
Cloning	NotITpCDS1xFlagR	AAT GCG GCC GCT CAC TTG TCG TCA TCG TCT TTG TAG TCG TAG TTG AAC TGG CG
Cloning	XbaITpCDS2xFlagR	CGT ATC TAG ATC ACT TAT CGT CGT CAT CCT TGT AAT CCT TAT CGT CGT CAT CCT TGT AAT CGT AGT TGA ACT GGC
Cloning	KpnITpCDSF	GAT CGG TAC CAT ATG TT CCA GCG CTT CAC C
RT-qPCR	qTpF1	GCA CCT CCT GAA GAC TCC AA
RT-qPCR	qTpR1	GGG TAA CAA ACC AGC TCT CG
RT-qPCR	qTpF2	GAA GCT GCC TTG CCT GAT C
RT-qPCR	qTpR2	CCG TTG CAA CAC TTT AGC AC
RT-qPCR	qTpF3	CCT CAT TGA GCA TCC CAG CA
RT-qPCR	qTpR3	CAG CTC TCC ATC GCT GAG G
RT-qPCR	qTpUTRF1	AAC CTT GTT GCT GTC CCA AG
RT-qPCR	qTpUTRR1	GAG CCA AAT GCC CTA TCA AA
RT-qPCR	qTpUTRF2	GGG CTC CTT CTT GCT GCT TA
RT-qPCR	qTpUTRR2	CCT GCT CCC TTC TGC TTC TC
RT-qPCR	qβActF1	ATG GAT GAC GAT ATC GCT GCG
RT-qPCR	qβActR1	GGT GAC AAT GCC GTG TTC AAT
RT-qPCR	qβActF2	TGT CCC TGT ATG CCT CTG GT
RT-qPCR	qβActR2	ATG TCA CGC ACG ATT TCC CT
RT-qPCR	qGFPF1	GAC GTA AAC GGC CAC AAG TT

RT-qPCR	qGFPR1	AAG TCG TGC TGC TTC ATG TG
RT-qPCR	qIVTF1	GTG TTA ACT TCC GAC TCC TCG C
RT-qPCR	qIVTR1	GTG ATG TCA AAC GAC GCA GC
RT-qPCR	qmCherryF1	GAG GGC ACC CAG ACC GCC AA
RT-qPCR	qmCherryR1	ACG CCG CCG TCC TCG AAG TT
RT-qPCR	qMsTpF1	GAG GAG GAT GAA GTG GAC GG
RT-qPCR	qMsTpR1	GGG AGG GGT AAC AAA CCA GC
RT-qPCR	qMsTpF2	GAG GAT GAA GTG GAC GGC TG
RT-qPCR	qMsTpR2	ACC AGC TTC TCG TCC ATC AAG
RT-qPCR	qMsTpF3	GAA GTG GAT GGC TGG CTC AT
RT-qPCR	qMsTpR3	TGC TGG GAT GCT CAA TGA GG
RT-qPCR	qHuTpUTRF1	CTC CCC TTC CTT CTG CCT TG
RT-qPCR	qHuTpUTRR1	TGC AGC TTC TCA ACG TGT CT
RT-qPCR	qHuTpUTRF2	TAG GGC CCC AGT AGT TGA CA
RT-qPCR	qHuTpUTRR2	GGC AGT GAG GAG TAG GCA AG
RT-qPCR	qGAPDHF1	CCT GCA CCA CCA ACT GCT TA
RT-qPCR	qGAPDHR1	CCA CGA TGC CAA AGT TGT CA
RT-qPCR	q18sF1	CGC CGC TAG AGG TGA AAT TC
RT-qPCR	q18sR1	TTG GCA AAT GCT TTC GCT C
