# Supplementary material for: Regulation of NGF Signaling by an Axonal Untranslated mRNA
Source: Neuron. 2019 May 8;102(3):553–563.e8. doi: 10.1016/j.neuron.2019.02.011 (PMC6509357; doi:10.1016/j.neuron.2019.02.011)
Supplement: Document S2. Article plus Supplemental Information [file mmc2.pdf]

Neuron

Report

Regulation of NGF Signaling by an Axonal Untranslated mRNA

Graphical Abstract

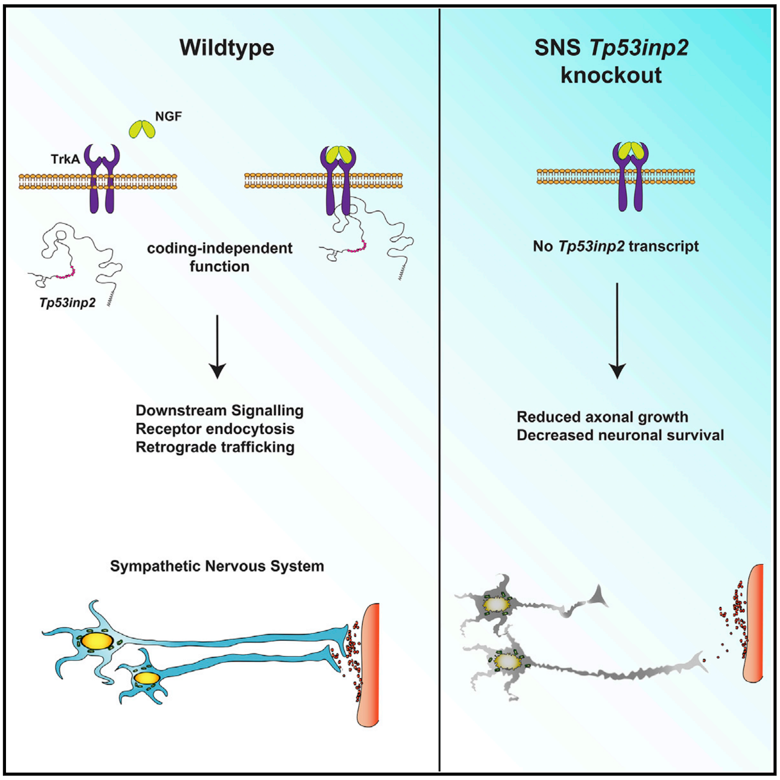

Highlights

- *Tp53inp2* is the most abundant mRNA in SN axons but is not translated
- *Tp53inp2* transcript interacts with the TrkA receptor to regulate NGF signaling
- In SNs, *Tp53inp2* functions independently of its protein-coding capacity
- *Tp53inp2* transcript is essential for axon growth and target innervation *in vivo*

Authors

Hamish Crerar, Emily Scott-Solomon, Chantal Bodkin-Clarke, ..., Marco Gaspari, Rejji Kuruvilla, Antonella Riccio

Correspondence

rkuruvilla@jhu.edu (R.K.), a.riccio@ucl.ac.uk (A.R.)

In Brief

Crerar et al. report that *Tp53inp2*, an mRNA transcript abundantly localized in axons of sympathetic neurons, interacts with the NGF receptor TrkA. The *Tp53inp2* transcript is not translated but acts in a coding-independent manner to regulate axon growth and neuronal survival *in vivo*.

Crerar et al., 2019, Neuron *102*, 553–563
May 8, 2019 © 2019 The Authors. Published by Elsevier Inc.
https://doi.org/10.1016/j.neuron.2019.02.011

CellPress

Regulation of NGF Signaling by an Axonal Untranslated mRNA

Hamish Crerar,[1,4] Emily Scott-Solomon,[2,4] Chantal Bodkin-Clarke,[2,4] Catia Andreassi,[1] Maria Hazbon,[2] Emilie Logie,[1] Marifé Cano-Jaimez,[1] Marco Gaspari,[3] Rejji Kuruvilla,[2,*] and Antonella Riccio[1,5,*]
[1]MRC Laboratory for Molecular Cell Biology, University College London, London, UK
[2]Department of Biology, The Johns Hopkins University, Baltimore, MD, USA
[3]Department of Experimental and Clinical Medicine, "Magna Græcia" University of Catanzaro, Catanzaro, Italy
[4]These authors contributed equally
[5]Lead Contact
*Correspondence: rkuruvilla@jhu.edu (R.K.), a.riccio@ucl.ac.uk (A.R.)
https://doi.org/10.1016/j.neuron.2019.02.011

SUMMARY

Neurons are extraordinarily large and highly polarized cells that require rapid and efficient communication between cell bodies and axons over long distances. In peripheral neurons, transcripts are transported along axons to growth cones, where they are rapidly translated in response to extrinsic signals. While studying *Tp53inp2*, a transcript highly expressed and enriched in sympathetic neuron axons, we unexpectedly discovered that *Tp53inp2* is not translated. Instead, the transcript supports axon growth in a coding-independent manner. Increasing evidence indicates that mRNAs may function independently of their coding capacity; for example, acting as a scaffold for functionally related proteins. The *Tp53inp2* transcript interacts with the nerve growth factor (NGF) receptor TrkA, regulating TrkA endocytosis and signaling. Deletion of *Tp53inp2* inhibits axon growth *in vivo*, and the defects are rescued by a non-translatable form of the transcript. *Tp53inp2* is an atypical mRNA that regulates axon growth by enhancing NGF-TrkA signaling in a translation-independent manner.

INTRODUCTION

Neurons are highly morphologically complex cells that require the expression of a large number of genes encoding proteins that support growth, branching and synaptic functions in dendrites, and growth cone migration, extension, and regeneration in axons (Andreassi et al., 2018; Terenzio et al., 2017). Neuronal cells rely on asymmetric localization of mRNAs to compartmentalize gene expression, a mechanism shared with most eukaryotic cells (Martin and Ephrussi, 2009). Transcripts are transported to dendrites and axons, where they can be rapidly translated in response to extrinsic signals, such as synaptic activity, neurotrophic factors, guidance cues, and injury (Andreassi et al., 2010; Cajigas et al., 2012; Gumy et al., 2011; Riccio, 2018; Sambandan et al., 2017; Terenzio et al., 2018). Genome-wide screens performed on RNA isolated from either dendrites or axons revealed that thousands of transcripts are asymmetrically localized in neurons (Andreassi et al., 2010, 2019; Cajigas et al., 2012; Gumy et al., 2011; Shigeoka et al., 2016). However, how peripherally localized transcripts influence axon growth and whether they may function independently of their translational capacity remains largely unknown.

Here we show that *Tp53inp2*, one of the most abundant and enriched mRNA transcripts in axons, is not translated in sympathetic neurons and regulates axon growth in a coding-independent manner. The unusually long 3′ UTR of *Tp53inp2* maintains the transcript in a translationally repressed state, possibly conferring to the transcript unique, neuron-specific roles. Importantly, we demonstrate that *Tp53inp2* interacts with the nerve growth factor (NGF) receptor TrkA, promoting receptor trafficking and intracellular signaling. Analysis of transgenic mice lacking *Tp53inp2* demonstrated that the gene is required for axon growth and sympathetic target innervation. Noticeably, the defects were rescued by a translation-deficient *Tp53inp2* transcript, indicating that, at least in sympathetic neurons, *Tp53inp2* functions independently of translation. Thus, our study reveals the essential role of the *Tp53inp2* transcript in regulating sympathetic neuron growth and innervation and represents the first evidence of an axonal mRNA capable of directly modulating NGF-TrkA signaling.

RESULTS AND DISCUSSION

The *Tp53inp2* Transcript Is Highly Expressed, but Not Translated, in Sympathetic Neuron Axons

Eukaryotic mRNAs include a coding sequence (CDS) encoding the protein and flanking UTRs of variable length, called 5′ and 3′ UTRs, that harbor regulatory elements that determine transcript localization, stability, and translation (Andreassi and Riccio, 2009; Lianoglou et al., 2013). To obtain a comprehensive characterization of the 3′ UTR transcript isoforms expressed in sympathetic neuron axons, we performed 3′ end RNA sequencing (RNA-seq) on mRNA isolated from either axons or cell bodies of rat sympathetic neurons cultured in compartmentalized chambers (Andreassi et al., 2019). In this model system, NGF is added only to the lateral axonal compartment, creating

© 2019 The Authors. Published by Elsevier Inc.
This is an open access article under the CC BY license (http://creativecommons.org/licenses/by/4.0/).

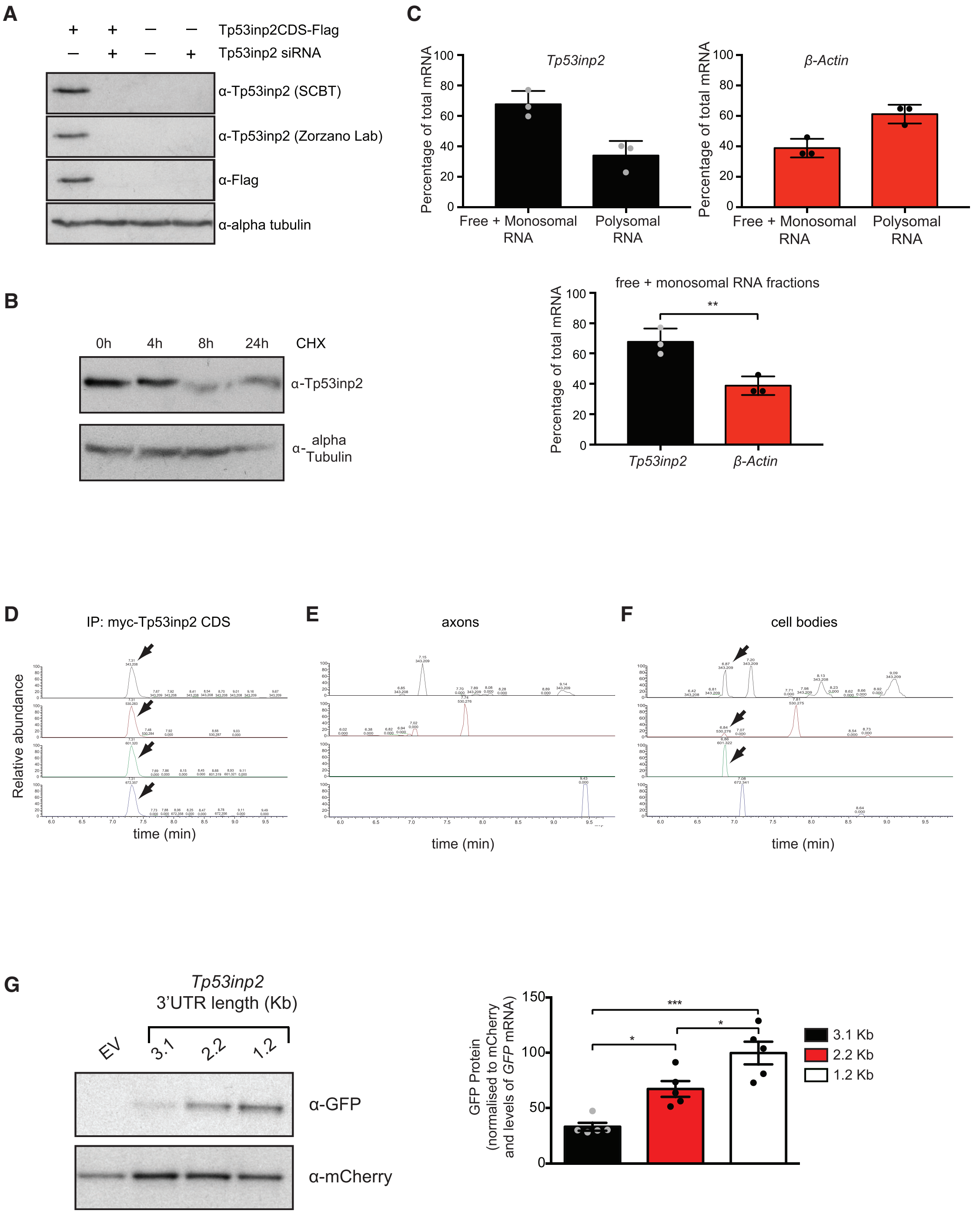

Figure 1. *Tp53inp2* Translation Is Repressed in Sympathetic Neurons

(A) Western blot of PC12 lysates transfected with Tp53inp2CDS-2xFLAG and Tp53inp2 siRNA, as indicated (n = 3).

(B) Western blot of lysates of HeLa cells treated with cycloheximide (CHX) for the indicated time (n = 3).

(C) qRT-PCR of *Tp53inp2* and *β-actin* in polysomal fractions from sympathetic neurons lysates; paired two-tailed t test (n = 3, **p < 0.01).

(D–F) Pseudo-selected reaction monitoring traces for the detection of a Tp53inp2 tryptic peptide in cultured sympathetic neuron axon (E) or cell body (F) samples and in an immunoprecipitated myc-Tp53inp2 control (D). The four traces represent the 4 most abundant fragments of the Tp53inp2 peptide ALHHAAAPMoxPAR. Arrows indicate where at least three transitions are detected at the same retention time, indicating peptide presence. Top value on trace, retention value; bottom value, mass to charge ratio (m/z).

(legend continued on next page)

experimental conditions that closely resemble the release of neurotrophins from target tissues (Kuruvilla et al., 2000; Riccio et al., 1997). mRNA was subjected to two rounds of linear poly(A) amplification before sequencing to enrich for 3′ UTRs (Andreassi et al., 2019; Andreassi et al., 2010). *Tp53inp2* was the most abundant transcript in axons, accounting for almost one-third of the reads (Figures S1A and S1B). The transcript is unusual in that the 3′ UTR is over 3,000 nt long (3,121 nt), accounting for nearly 80% of the transcript length, whereas the open reading frame (ORF) is 666 nt long, encoding a small protein of predicted low complexity. Although the Tp53inp2 protein has been implicated in the regulation of autophagy in skeletal muscle fibers and other mammalian cell lines (Nowak et al., 2009; Sala et al., 2014), extensive attempts to detect the endogenous Tp53inp2 protein in PC12 cells and sympathetic neurons using either homemade, commercial, or previously published antibodies were unsuccessful. Western blotting of PC12 cells transfected with a vector expressing the CDS of *Tp53inp2* showed that, under these conditions, the transcript was translated and easily detected (Figure 1A; Figures S1C and S1D). Co-transfection with a small interfering RNA (siRNA) that efficiently inhibited *Tp53inp2* expression completely abolished the signal (Figure 1A), indicating the specificity of the antibodies. Importantly, we tested several cell types and confirmed that endogenous Tp53inp2 was expressed in HeLa cells (Xu et al., 2016) and that the protein was stable, with a half-life of at least 4 h (Figure 1B; Figure S1E).

To further investigate whether *Tp53inp2* mRNA was translated in sympathetic neurons, we performed polysome fractionation (Johannes and Sarnow, 1998). Although not devoid of limitations, the technique allows an accurate prediction of transcript translation rates. qRT-PCR of the *Tp53inp2* transcript associated with either polyribosomes or monosomes revealed that, in sympathetic neurons, a large fraction of *Tp53inp2* mRNA co-sedimented with the monosomal fraction, whereas the efficiently translated transcript *β-actin* was mostly associated with polysomes (Figure 1C; Figure S1F). Because monosomes in yeast can translate transcripts containing an ORF of up to 600 nt (Heyer and Moore, 2016), we further investigated, using mass spectrometry, whether Tp53inp2 protein was expressed in sympathetic neurons. Three proteotypic peptides identified by a data-dependent analysis of immunoprecipitated Tp53inp2 were selected for high-sensitivity detection in targeted mode (Table S1). Targeted mass spectrometry performed either on severed axons or cell bodies of sympathetic ganglia explants (superior cervical ganglia [SCG]) confirmed that Tp53inp2 peptides were not present in axons (Figures 1D–1F; Figure S1G). Very small signals corresponding to ALHHAAAPMoxPAR and HQGSFIYQPCQR fragment ions were detected at the limit of the signal-to-noise ratio only in cell bodies (Figure 1F; Figure S1G). It should be noted however, that SCG explants contain few non-neuronal cells that may account for the Tp53inp2 peptides detected in the cell body samples. Thus, although we cannot exclude the possibility that the *Tp53inp2* transcript is translated at extremely low levels in cell bodies of sympathetic neurons, Tp53inp2 protein was undetectable in axons using three distinct sensitive technical approaches.

The unexpected finding that, despite its abundance, *Tp53inp2* mRNA was not synthesized into a protein prompted us to investigate the mechanisms that maintain the transcript in a translationally silent state in sympathetic neurons. Because vectors expressing only the coding region of *Tp53inp2* were translated (Figure 1A; Figures S1C and S1D), we reasoned that the *Tp53inp2* 3′ UTR may harbor regulatory elements that inhibit translation. To this end, we generated expression vectors carrying the 5′ UTR of *Tp53inp2* upstream of the coding region of GFP and followed by either the full-length (~3,100 nt) or truncated forms (~2,200 nt and ~1,200 nt, respectively) of the *Tp53inp2* 3′ UTR. Although there were some differences in the expression levels of one vector (Figure S1I), when relative mRNA expression was taken into account, shortening of the 3′ UTR correlated with substantial relief of translational inhibition (Figure 1G; Figure S1H). A possible implication of these results is that cells expressing Tp53inp2 protein also express a *Tp53inp2* transcript harboring a shorter 3′ UTR. Indeed, qRT-PCR performed on RNA isolated from either sympathetic neurons or HeLa cells revealed that an isoform bearing a shorter 3′ UTR was predominantly expressed in HeLa cells (Figure S1J). These findings indicate that elements contained within the longer 3′ UTR may be responsible for maintaining the transcript translationally silent in sympathetic neurons.

The *Tp53inp2* Transcript Interacts with the TrkA Receptor to Mediate NGF Signaling

Recent studies have shown that 3′ UTRs may have a more flexible role in regulating gene expression than previously thought (Andreassi et al., 2018; Mayr, 2017). In hippocampal neurons, for example, an E3 ubiquitin ligase isoform expressing a short 3′ UTR (*Ube3a1*) inhibits dendritogenesis in a coding-independent manner by competing with endogenous *Ube3a* for miR-134 binding (Valluy et al., 2015). Moreover, in cancer cell lines, the long 3′ UTR of the *CD47* transcript acts as a scaffold for RNA binding proteins (RBPs) and determines the translocation of the CD47 protein to the plasma membrane (Berkovits and Mayr, 2015).

In axons, NGF binds to its cognate receptor TrkA and, following receptor dimerization and autophosphorylation, ligand-receptor complexes are internalized within endosomes and retrogradely transported to the cell bodies (Harrington and Ginty, 2013; Yamashita and Kuruvilla, 2016). Signaling endosomes regulate axon growth and are transported long-distance to somato-dendritic compartments to modulate synapse assembly and activate transcription (Lehigh et al., 2017; Scott-Solomon and Kuruvilla, 2018). Given the lack of translation, we reasoned that in sympathetic neurons, the *Tp53inp2* transcript could influence NGF signaling in a coding-independent manner. We first investigated

(G) Left: western blot of PC12 cells co-transfected with GFP fusion constructs containing *Tp53inp2* 3′ UTR 3.1, 2.2, or 1.2 kb and an mCherry control vector. Right: densitometry of GFP protein levels was normalized by mCherry levels and then further normalized by levels of *GFP* mRNA. Values are expressed as percentage of the mean GFP protein amount of the 1.2-kb construct. Ordinary one-way ANOVA, Tukey's multiple comparisons test (n = 5, *p < 0.05, ***p < 0.001). Data are presented as average ± SEM. See also Figure S1.

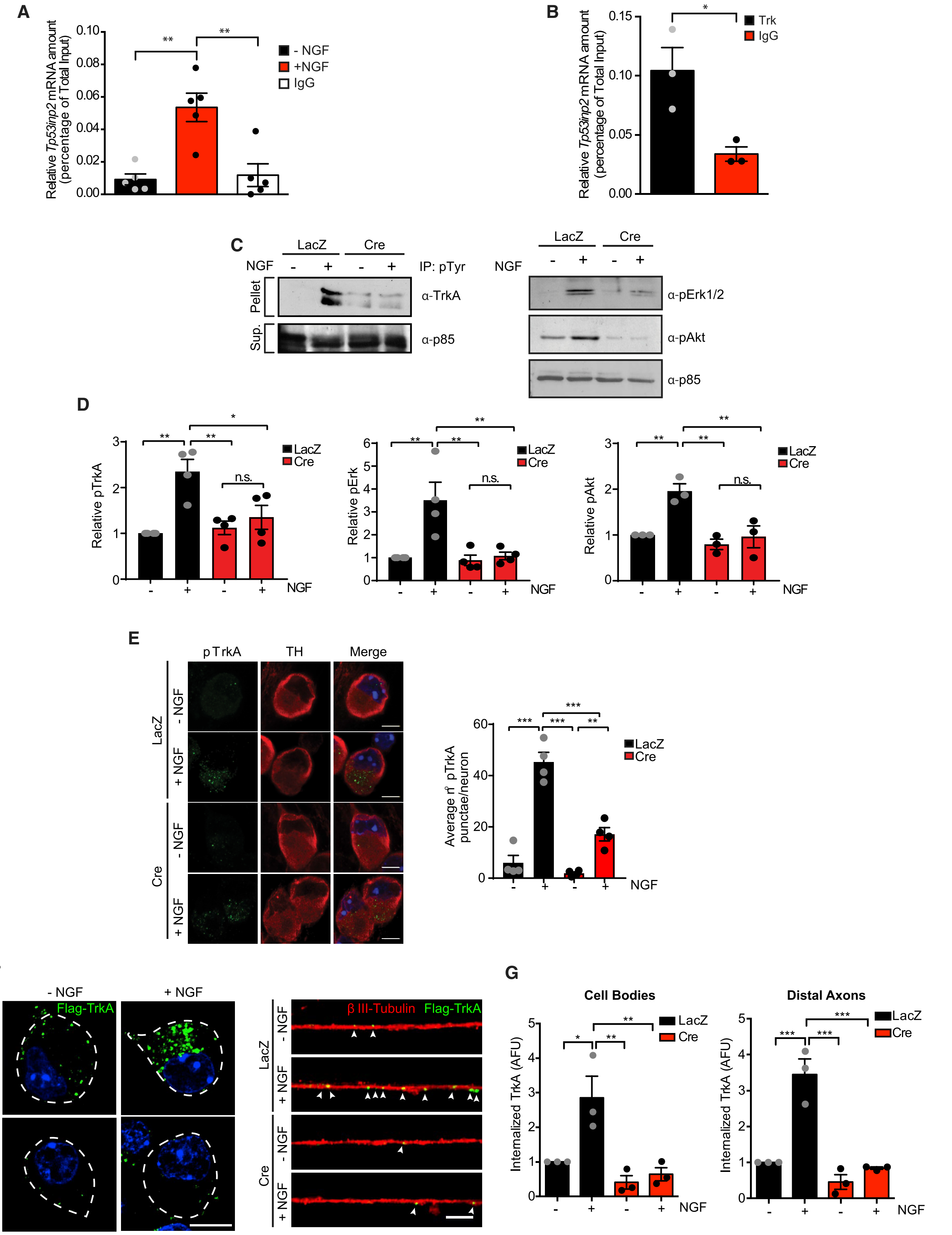

(legend on next page)

whether *Tp53inp2* transcripts interacted with the TrkA receptor in axons by performing RNA immunoprecipitation (RIP) on sympathetic neurons. Remarkably, the pan-Trk antibody immunoprecipitated the *Tp53inp2* transcript, and the interaction was greatly increased in response to NGF stimulation (Figure 2A; Figure S2A). The cognate receptor TrkB also interacted with *Tp53inp2* in mouse cortical neurons (Figure 2B), whereas, in sympathetic neurons, the highly abundant axonal transcript *IMPA1-L* was not immunoprecipitated (Andreassi et al., 2010; Figure S2A). Both transcripts were immunoprecipitated by the neuron-specific RBP HuD (Figures S2B and S2C), and endogenous HuD and TrkA co-immunoprecipitated in sympathetic neurons (Figure S2D), suggesting that HuD may mediate TrkA binding to the *Tp53inp2* transcript. As a control, we performed RIP of *Tp53inp2* with another transmembrane protein, NCAM, and found no significant interaction (Figures S2E and S2F).

Next, we asked whether the *Tp53inp2* transcript influences NGF signaling in sympathetic neurons. To this end, we generated transgenic mice carrying a floxed allele of *Tp53inp2*, where exons 2 and 3 are flanked by *loxP* sites (*Tp53inp2$^{fl/fl}$* mice) (Figure S2G). To assess the effects of *Tp53inp2* loss on NGF signaling in axons, SCG explants isolated from *Tp53inp2$^{fl/fl}$* mice on postnatal day 0.5 (P0.5) were infected with either an adenoviral vector expressing the Cre recombinase to acutely delete *Tp53inp2* (Figure S2H) or a LacZ virus as a control. Cell bodies were mechanically severed, and the isolated axons were stimulated with NGF. Axon protein lysate was either immunoblotted for the canonical signaling effectors Erk1-2 and Akt or subjected to immunoprecipitation with a phospho-tyrosine antibody, and the pellet was immunoblotted using antibodies for TrkA. As expected, NGF stimulation increased pTrkA, pAkt, and pErk1-2 levels in axons under control conditions, whereas NGF-mediated phosphorylation of TrkA and downstream effectors was inhibited in axons lacking *Tp53inp2* (Figures 2C and 2D), indicating that *Tp53inp2* interaction with TrkA receptors mediates TrkA signaling in axons. Moreover, immunofluorescence analysis showed that *Tp53inp2* depletion significantly decreased the retrograde appearance of phosphorylated TrkA receptors in cell bodies of cultured sympathetic neurons lacking *Tp53inp2* (Figure 2E).

A key event that mediates NGF-Trk signaling is the ligand-induced endocytosis of TrkA receptors. The inhibition of local and retrograde NGF signaling in *Tp53inp2*-deficient neurons prompted us to investigate whether *Tp53inp2* could influence TrkA internalization. To this end, we performed a live-cell antibody-feeding assay (Yamashita et al., 2017) on *Tp53inp2$^{fl/fl}$* sympathetic neurons co-infected with adenoviruses expressing either LacZ or Cre and FLAG-TrkA. Robust receptor internalization was observed in both cell bodies and axons of control neurons in response to NGF stimulation (Figures 2F and 2G). In contrast, internalization was markedly attenuated in neurons lacking *Tp53inp2*. Similar results were obtained when internalization of endogenous TrkA receptors was probed using a cell surface biotinylation assay (Houtz et al., 2016). NGF-induced TrkA internalization was significantly reduced in neurons lacking *Tp53inp2* compared with control conditions (Figure S3A). Loss of *Tp53inp2* had no effect on basal levels of surface TrkA (Figures S3B and S3C) or on total TrkA protein expression in sympathetic neurons (Figure S3D). Together, these results indicate that *Tp53inp2* regulates NGF-dependent TrkA internalization.

Tp53inp2 Is a Translationally Repressed mRNA that Affects Cell Survival and Axon Growth

The findings that the *Tp53inp2* transcript interacts with the TrkA receptor and regulates NGF signaling in sympathetic neurons prompted us to investigate whether it affected NGF-dependent axon growth and cell survival. *Tp53inp2$^{fl/fl}$* sympathetic neurons were grown in compartmentalized chambers with NGF added only to distal axons and infected with either Cre or LacZ adenoviruses (Figure S3E). Neurons expressing LacZ showed robust growth in the presence of NGF (Figures 3A and S3B). In contrast, NGF-mediated growth was remarkably stunted in neurons lacking *Tp53inp2* (Figures 3A and S3B). To determine whether the

Figure 2. *Tp53inp2* mRNA Interacts with the TrkA Complex to Regulate NGF Signaling and Endocytosis in Sympathetic Neurons

(A) RNA immunoprecipitation (RIP) performed on sympathetic neuron lysates using antibodies for pan-Trk (sympathetic neurons grown under NGF stimulation predominantly express TrkA) or IgG. The levels of *Tp53inp2* were analyzed by qRT-PCR. Ordinary one-way ANOVA, Tukey's multiple comparisons test (n = 5; $^{**}p < 0.01$).

(B) RIP performed on mouse cortical neuron lysates using antibodies for pan-Trk (cortical neurons predominantly express TrkB) or IgG. The levels of *Tp53inp2* mRNA were analyzed by qRT-PCR. Unpaired two-tailed t test (n = 3, $^{*}p < 0.05$).

(C) Western blot of axonal lysates from *Tp53inp2$^{fl/fl}$* SCG explants infected with an adenovirus expressing either Cre or LacZ. Isolated axons were stimulated with NGF or left untreated before immunoprecipitation with a phospho-tyrosine (PY20) antibody, followed by immunoblotting for TrkA (pellet). Supernatants (Sup.) were immunoblotted as indicated.

(D) Densitometry analysis of data shown in (C). Values are normalized relative to the no neurotrophin condition for LacZ-expressing neurons. Ordinary two-way ANOVA, Tukey's multiple comparisons test (n = 3–4, $^{*}p < 0.05$, $^{**}p < 0.01$).

(E) Left: representative images of pTrkA and tyrosine hydroxylase (TH) immunostaining in cell bodies of *Tp53inp2$^{fl/fl}$* sympathetic neurons infected with an adenovirus expressing either Cre or LacZ. Distal axons were stimulated with NGF or left unstimulated. Scale bar, 5 μm. Right: quantification of pTrkA puncta per neuron. Ordinary two-way ANOVA, Tukey's multiple comparisons test (n = 4; each data point represents the average of 20–30 neurons per condition per experiment; $^{***}p < 0.001$, $^{**}p < 0.01$).

(F) Representative images of FLAG-TrkA immunostaining in cell bodies (left) and axons (right) of *Tp53inp2$^{fl/fl}$* neurons infected with an adenovirus expressing LacZ or Cre and co-infected with a FLAG-TrkA adenovirus. Neurons were live-labeled with FLAG antibody under non-permeabilizing conditions and NGF-treated as indicated. Arrows indicate internalized FLAG-TrkA receptors in axons. Scale bar, 5 μm.

(G) Quantification of the data in (F). Average fluorescence density was determined per square micrometer for each cell body or per micrometer for each axon. Values are expressed relative to no NGF condition in LacZ-expressing neurons. Data are presented as average fluorescence density ± SEM. Two-way ANOVA (n = 3, $^{*}p < 0.05$, $^{**}p < 0.01$, $^{***}p < 0.001$). At least 30–50 cell bodies and 20–30 axons were analyzed per condition.

Data are presented as average ± SEM. See also Figure S2.

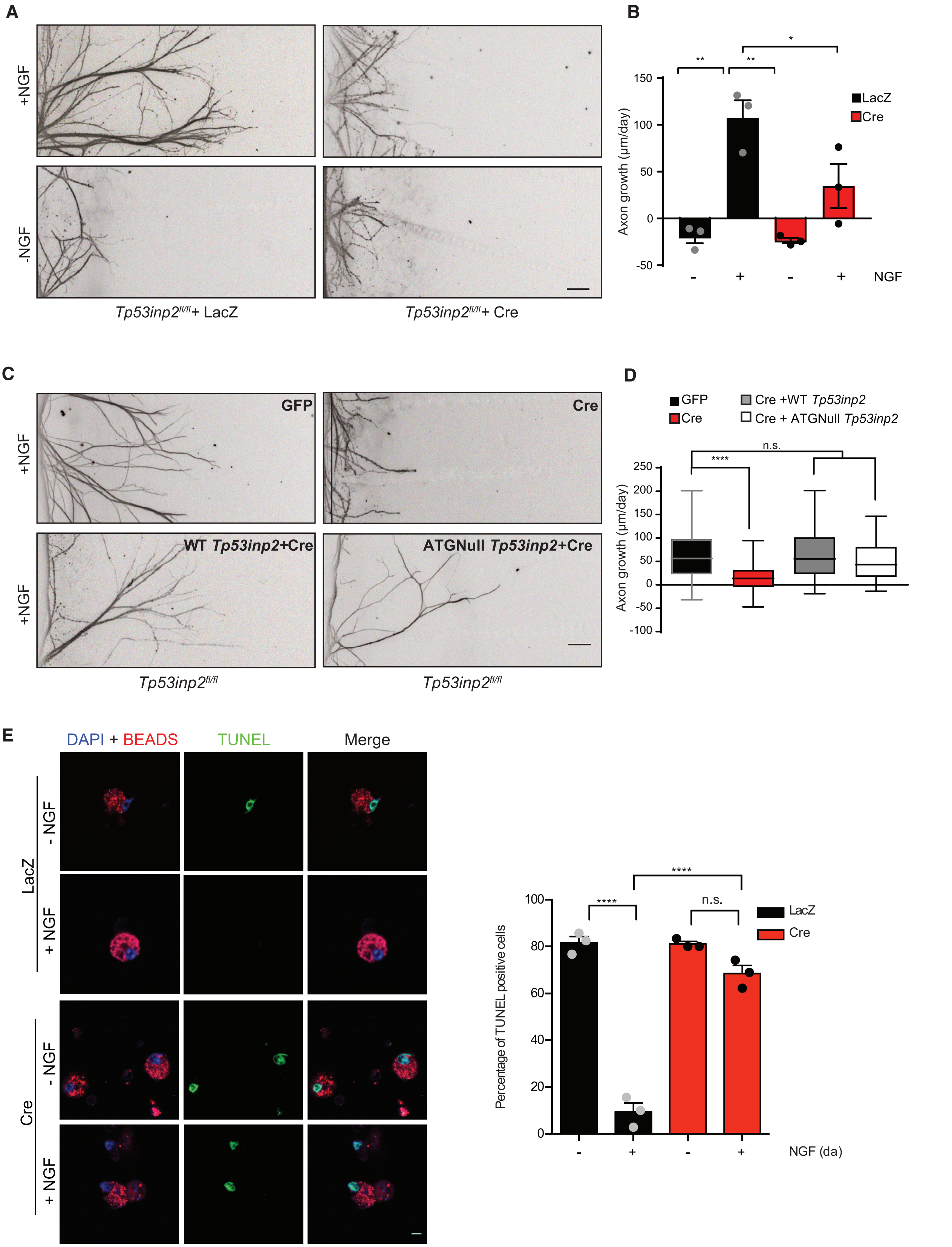

(legend on next page)

Tp53inp2 transcript rescued the growth defects induced by *Tp53inp2* loss, adenoviral vectors expressing either full-length wild-type (WT) *Tp53inp2* or a form bearing mutations of all putative translational start sites within the CDS (ATGNull) were generated (Figure S3F). Western blotting and qRT-PCR confirmed that the WT and ATGNull *Tp53inp2* transcripts were not translated despite being transcribed efficiently in PC12 cells (Figures S3G, S4A, and S4B). When *Tp53inp2*-deficient neurons were infected with either one of the two adenoviral vectors, we observed that the WT and ATGNull *Tp53inp2* were equally efficient in rescuing axon growth defects induced by the loss of *Tp53inp2* (Figures 3C and 3D). Together, these findings indicate that *Tp53inp2* translation is not required for promoting sympathetic neuron axon growth and support the hypothesis that it may function outside of its coding capacity.

Next, we asked whether *Tp53inp2* loss would compromise the ability of axonally applied NGF to retrogradely support neuronal survival. The survival of *Tp53inp2*$^{fl/fl}$ neurons was studied in the absence or presence of NGF on distal axons. When NGF was withdrawn, 80% of neurons in both LacZ- and Cre-infected neurons underwent apoptotic cell death (Figure 3E). NGF added to the distal axons was sufficient to promote survival of the majority of LacZ-infected neurons, whereas neurons lacking *Tp53inp2* continued to exhibit significant levels of apoptosis (Figure 3E; Figure S4C). In line with evidence showing that NGF applied directly to cell bodies promotes neuronal survival by an endocytosis-independent mechanism (Riccio et al., 1997; Ye et al., 2003), we found that, under these conditions, loss of *Tp53inp2* did not increase apoptosis (Figure 3E; Figure S4C). Thus, *Tp53inp2* supports neuronal survival by regulating endocytosis and retrograde trafficking of NGF-TrkA receptor complexes.

The *Tp53inp2* Transcript Is Necessary for Sympathetic Neuron Target Innervation

Next, we investigated whether the loss of *Tp53inp2* affected sympathetic neuron survival and growth *in vivo*. First, *in situ* hybridization confirmed that *Tp53inp2* transcript is expressed in sympathetic neuron ganglia at high levels from embryonic day 14.5 (E14.5) until early postnatal stages, a time when they are highly dependent on NGF for cell survival and axon growth (Figure 4A). Fluorescence *in situ* hybridization (FISH) of the 3′ UTR of the *Tp53inp2* transcript showed punctate staining in both axons and cell bodies of sympathetic neurons (Figure 4B). The FISH signal was lost when the assay was performed on *Tp53inp2*$^{fl/fl}$ neurons infected with an adenoviral vector expressing the Cre recombinase, confirming the specificity of the FISH signal.

To generate mice lacking *Tp53inp2* in sympathetic neurons, *Tp53inp2*$^{fl/fl}$ animals were crossed with transgenic mice expressing the Cre recombinase under the control of the tyrosine hydroxylase promoter (*Th-Cre;Tp53inp2*$^{fl/fl}$ mice; Gong et al., 2007; Figure S2G), which resulted in efficient deletion (Figure S4D). Analyses of the SCGs revealed normal numbers of sympathetic neurons in *Th-Cre;Tp53inp2*$^{fl/fl}$ mice at E16.5 but significant cell loss at birth (P0.5) and 3 weeks after birth (P21) compared with control littermates (Figure 4C). Despite the absence of neuronal loss at E16.5, sympathetic innervation of the heart was decreased in the mutant embryos, with a significant reduction in both density of innervation and axonal branching in *Th-Cre;Tp53inp2*$^{fl/fl}$ embryos compared with control littermates (Figures 4D and 4E). Taken together, these findings indicate that *Tp53inp2* is required for the development of sympathetic neurons when they are most dependent on NGF for cell survival and axon growth.

NGF is secreted by the target tissues and acts locally in axons to support growth. The signal generated by the interaction of NGF with TrkA receptors is also retrogradely transported to the cell bodies, where it initiates nuclear events necessary for cell survival and axon growth (Ascano et al., 2012; Harrington and Ginty, 2013). We observed that the defects of target innervation preceded neuronal loss, suggesting that lack of *Tp53inp2* primarily affects axon growth *in vivo*. Thus, in mice lacking *Tp53inp2*, neuronal loss was likely due to the axons failing to reach the target tissues and gain access to adequate levels of NGF. We cannot exclude, however, that impaired retrograde NGF signaling from distal axons to the cell bodies may also play a role.

Crosstalk between NGF Signaling and mRNA Transcripts in Axons

Neurotrophic signaling is essential for the development of the sympathetic nervous system. The binding of neurotrophins to Trk receptors induces a variety of cellular processes required

Figure 3. *Tp53inp2* Function in Axon Growth Is Translation Independent

(A) Compartmentalized cultures of *Tp53inp2*$^{fl/fl}$ sympathetic neurons infected with an adenovirus expressing LacZ or Cre were either maintained with NGF in axons or deprived of NGF. Shown are representative images of axons immunostained with anti-β-III tubulin 48 h after starting the treatments. Scale bar, 100 μm.

(B) Average growth rate of axons measured at 24-h intervals for 72 h. Ordinary two-way ANOVA, Tukey's multiple comparisons test (n = 3, each data point represents the average at least 70 axons traced per condition, $^{**}p < 0.01$, $^{*}p < 0.05$).

(C) *Tp53inp2*$^{fl/fl}$ sympathetic neurons cultured in compartmentalized chambers were infected with adenoviruses as indicated. Shown are representative images of axons immunostained with anti-β-III tubulin 48 h after infection. Scale bar, 100 μm.

(D) Average growth rate of axons at 24 h intervals for a total of 72 h. Ordinary two-way ANOVA, Dunnett's multiple comparisons test (n = 3, each data point represents the average of at least 50 axons traced per condition; hinges correspond to the first and third quartiles, the center line corresponds to the median, and the maxima and minima correspond to 5–95 percentiles; $^{****}p < 0.0001$).

(E) Left: distal axons of *Tp53inp2*$^{fl/fl}$ neurons infected with an adenovirus expressing LacZ or Cre were stimulated with NGF or deprived of NGF. Neurons that had projected to axonal chambers were identified through the uptake of inert fluorescent beads (red) supplied to the axon chambers. Neuronal apoptosis was detected using terminal deoxynucleotidyl transferase dUTP nick end labelling (TUNEL) staining. Scale bar, 5 μm. Right: quantification of neuronal cell death. Ordinary two-way ANOVA, Tukey's multiple comparisons test (n = 3, each data point represents the average of at least 30 cells per condition per experiment, $^{****}p < 0.0001$).

Data are presented as average ± SEM. See also Figures S3 and S4.

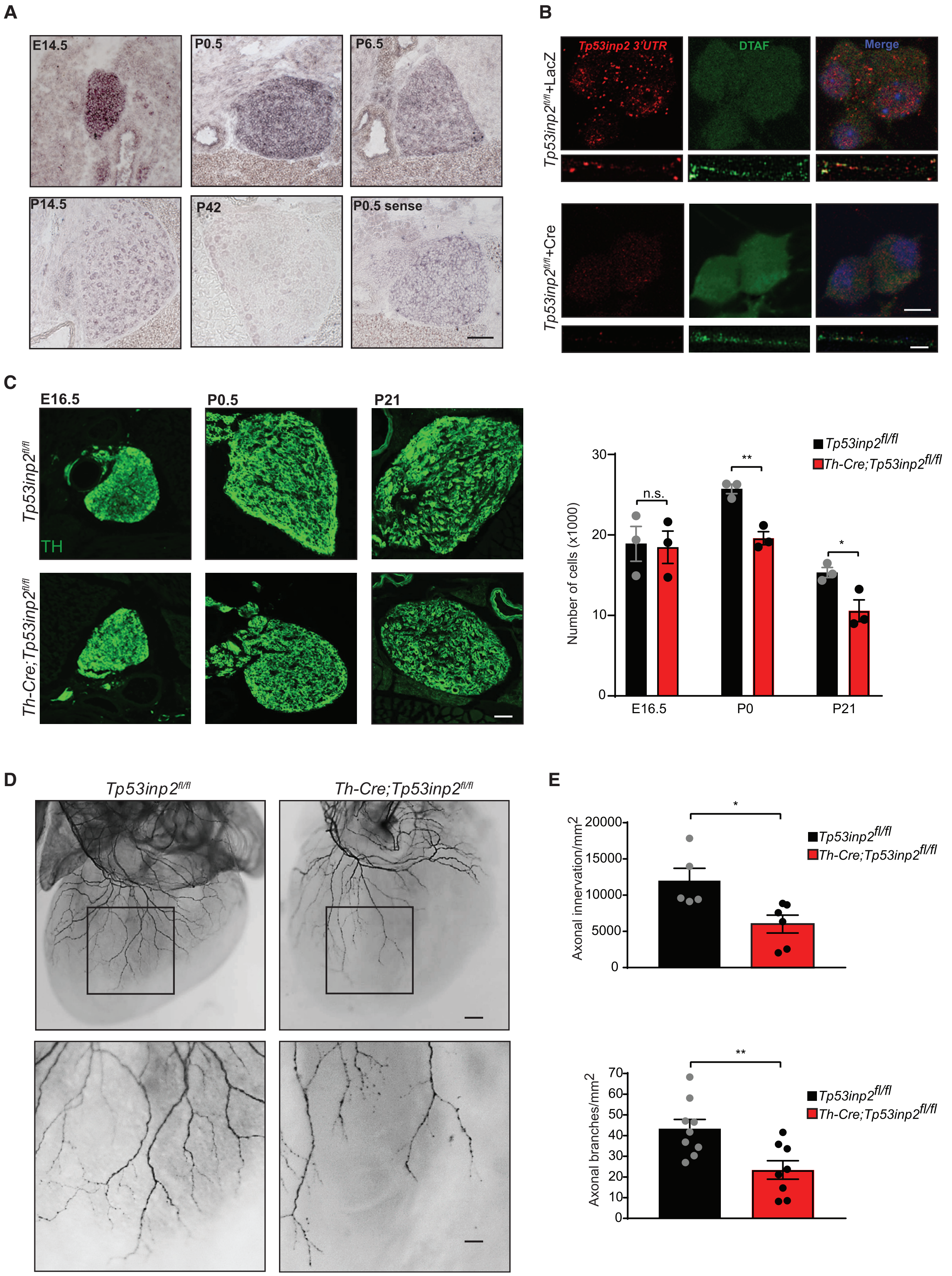

(legend on next page)

for survival, axon growth, dendritogenesis, and synaptogenesis (Scott-Solomon and Kuruvilla, 2018), which are principally mediated by transcriptional activation and extensive RNA localization in dendrites and axons (Andreassi et al., 2010, 2019; Cosker et al., 2016). Although it is well known that neurotrophic signaling affects gene transcription, asymmetric localization of RNA, and local translation, whether the opposite event may occur was unknown.

Our findings add to the small but growing list of mRNA transcripts that, in addition to being translated into proteins, harbor coding-independent functions (Nam et al., 2016), such as acting as a sponge for miRNA (Valluy et al., 2015) or as a scaffold for the formation of protein complexes (Berkovits and Mayr, 2015). Importantly, the finding that *Tp53inp2* mRNA acts in a coding-independent manner in neurons but is translated in other cell types adds a further layer of complexity to RNA regulation and provides evidence for a new mechanism that exploits the 3′ UTR to confer cell-type-specific functions to ubiquitously expressed transcripts.

STAR★METHODS

Detailed methods are provided in the online version of this paper and include the following:

- KEY RESOURCES TABLE
- CONTACT FOR REAGENT AND RESOURCE SHARING
- EXPERIMENTAL MODEL AND SUBJECT DETAILS
  - Generation of Tp53inp2$^{fl/fl}$ mutant mice
  - Mouse husbandry
  - Primary cultures
  - Mouse cortical neurons
  - PC12 cell line
  - HEK293 cell line
  - HeLa cell line
- METHOD DETAILS
  - Adenoviral vectors and Plasmids
  - RNA isolation and RT-qPCR
  - *In situ* hybridization
  - smFISH
  - Neuronal counts
  - Whole-mount diaminobenzidine-TH immunohistochemistry
  - Neuronal survival
  - Axon growth
  - Polysomal Fractionation
  - Mass Spectrometry
  - NanoLC-MS/MS analysis
  - RNA Immunoprecipitation
  - Immunoprecipitation and immunoblotting analyses
  - pTrkA Immunostaining
  - Analyses of surface TrkA, receptor endocytosis, and total TrkA
- QUANTIFICATION AND STATISTICAL ANALYSIS
- DATA AND SOFTWARE AVAILABILITY

SUPPLEMENTAL INFORMATION

Supplemental Information includes four figures and two tables and can be found with this article online at https://doi.org/10.1016/j.neuron.2019.02.011.

ACKNOWLEDGMENTS

We thank C. Gerfen (NIH, USA) for the kind gift of the TH-Cre mice, R. Luisier (The Francis Crick Institute, London) for providing the 3′ RNA-seq track for *Tp53inp2* in Figure S1B, and A. Zorzano (IRB, Barcelona) for the generous gift of the Tp53inp2 antibody. We thank Adolfo Saiardi, Alison Lloyd, Jernej Ule, Haiqing Zhao, and Samer Hattar for insightful suggestions regarding the manuscript and all members of the Riccio and Kuruvilla labs for helpful discussions. This work was supported by Wellcome Trust investigator award 103717/Z/14/Z (to A.R.), MRC senior non-clinical fellowship SNCF G0802010 (to A.R.), MRC LMCB core grant MC_U12266B, Wellcome Trust institutional strategic support fund 2014 (to C.A.), NIH R01 DK108267 and NS073751 (to R.K.), NIH training grant T32GM007231 (to C.B.-C. and E.S-S.), and MIUR, Programma Operativo Nazionale, iCARE Project grant ICARE PON03PE_0009_2 (to M.G.).

AUTHOR CONTRIBUTIONS

H.C., E.S.-S., C.B.-C., A.R., and R.K. conceived the work. H.C., C.B.-C., E.S.-S., C.A., E.L., M.H., and M.C.-J. performed the experiments. M.G. performed the mass spectrometry analysis. A.R. and R.K. wrote the manuscript with input from all authors.

DECLARATION OF INTERESTS

The authors declare no competing interests.

Received: April 5, 2018
Revised: November 26, 2018
Accepted: February 6, 2019
Published: March 7, 2019

Figure 4. *Tp53inp2* Is Essential for the Growth of NGF-Responsive Sympathetic Neurons

(A) *In situ* hybridization of *Tp53inp2* mRNA in the developing mouse SCG at the indicated developmental stages. Hybridization of the control sense probe is shown at P0.5. Scale bar, 100 μm.

(B) Fluorescence *in situ* hybridization (FISH) of the *Tp53inp2* 3′ UTR in cell bodies (top) and axons (bottom) of adenovirus-infected *Tp53inp2$^{fl/fl}$* sympathetic neurons. Scale bars, cell bodies, 10 μm; axons, 20 μm.

(C) TH immunohistochemistry of SCGs from *Th-Cre;Tp53inp2$^{fl/fl}$* and control *Tp53inp2$^{fl/fl}$* mice at the indicated developmental stages (left). Counts of cell number were performed on Nissl-stained tissue sections (right). Scale bar, 200 μm. Unpaired t test (n = 3 mice per genotype, $*p < 0.05$, $**p < 0.01$).

(D) Whole-mount TH immunostaining of the heart in E16.5 *Th-Cre;Tp53inp2$^{fl/fl}$* mice and *Tp53inp2$^{fl/fl}$* control littermates. Higher magnification images of the boxed area are shown at the bottom. Scale bars, 100 μm (top) and 400 μm (bottom).

(E) Quantitative analysis of the data shown in (D). Total innervation was measured as the area covered by TH-positive axon fibers (top, n = 5 and 6 per *Tp53inp2$^{fl/fl}$* and *Th-Cre;Tp53inp2$^{fl/fl}$*). The branchpoints were counted as the number of axon terminal endpoints (bottom, n = 9 and 8 per *Tp53inp2$^{fl/fl}$* and *Th-Cre;Tp53inp2$^{fl/fl}$*). Unpaired two-tailed t test ($*p < 0.05$, $**p < 0.01$).

Data are presented as average ± SEM. See also Figure S4.

REFERENCES

Andreassi, C., and Riccio, A. (2009). To localize or not to localize: mRNA fate is in 3'UTR ends. Trends Cell Biol. *19*, 465–474.

Andreassi, C., Zimmermann, C., Mitter, R., Fusco, S., De Vita, S., Saiardi, A., and Riccio, A. (2010). An NGF-responsive element targets myo-inositol monophosphatase-1 mRNA to sympathetic neuron axons. Nat. Neurosci. *13*, 291–301.

Andreassi, C., Crerar, H., and Riccio, A. (2018). Post-transcriptional processing of mRNA in neurons: the vestiges of the RNA world drive transcriptome diversity. Front. Mol. Neurosci. *11*, 304.

Ascano, M., Richmond, A., Borden, P., and Kuruvilla, R. (2009). Axonal targeting of Trk receptors via transcytosis regulates sensitivity to neurotrophin responses. J. Neurosci. *29*, 11674–11685.

Andreassi, C., Luisier, R., Crerar, H., Franke, S., Luscombe, M., Cuda, G., Gaspari, M., and Riccio, A. (2019). 3′UTR cleavage of transcripts localized in axons of sympathetic neurons. bioRxiv. https://doi.org/10.1101/170100.

Ascano, M., Bodmer, D., and Kuruvilla, R. (2012). Endocytic trafficking of neurotrophins in neural development. Trends Cell Biol. *22*, 266–273.

Baumgartner, B.G., Orpinell, M., Duran, J., Ribas, V., Burghardt, H.E., Bach, D., Villar, A.V., Paz, J.C., González, M., Camps, M., et al. (2007). Identification of a novel modulator of thyroid hormone receptor-mediated action. PLoS ONE *2*, e1183.

Berkovits, B.D., and Mayr, C. (2015). Alternative 3′ UTRs act as scaffolds to regulate membrane protein localization. Nature *522*, 363–367.

Bodmer, D., Ascano, M., and Kuruvilla, R. (2011). Isoform-specific dephosphorylation of dynamin1 by calcineurin couples neurotrophin receptor endocytosis to axonal growth. Neuron *70*, 1085–1099.

Cajigas, I.J., Tushev, G., Will, T.J., tom Dieck, S., Fuerst, N., and Schuman, E.M. (2012). The local transcriptome in the synaptic neuropil revealed by deep sequencing and high-resolution imaging. Neuron *74*, 453–466.

Chen, K.H., Boettiger, A.N., Moffitt, J.R., Wang, S., and Zhuang, X. (2015). RNA imaging. Spatially resolved, highly multiplexed RNA profiling in single cells. Science *348*, aaa6090.

Cosker, K.E., Fenstermacher, S.J., Pazyra-Murphy, M.F., Elliott, H.L., and Segal, R.A. (2016). The RNA-binding protein SFPQ orchestrates an RNA regulon to promote axon viability. Nat. Neurosci. *19*, 690–696.

Gong, S., Doughty, M., Harbaugh, C.R., Cummins, A., Hatten, M.E., Heintz, N., and Gerfen, C.R. (2007). Targeting Cre recombinase to specific neuron populations with bacterial artificial chromosome constructs. J. Neurosci. *27*, 9817–9823.

Green, S.H., Rydel, R.E., Connolly, J.L., and Greene, L.A. (1986). PC12 cell mutants that possess low- but not high-affinity nerve growth factor receptors neither respond to nor internalize nerve growth factor. J. Cell Biol. *102*, 830–843.

Gumy, L.F., Yeo, G.S., Tung, Y.C., Zivraj, K.H., Willis, D., Coppola, G., Lam, B.Y., Twiss, J.L., Holt, C.E., and Fawcett, J.W. (2011). Transcriptome analysis of embryonic and adult sensory axons reveals changes in mRNA repertoire localization. RNA *17*, 85–98.

Harrington, A.W., and Ginty, D.D. (2013). Long-distance retrograde neurotrophic factor signalling in neurons. Nat. Rev. Neurosci. *14*, 177–187.

Heyer, E.E., and Moore, M.J. (2016). Redefining the translational status of 80S monosomes. Cell *164*, 757–769.

Houtz, J., Borden, P., Ceasrine, A., Minichiello, L., and Kuruvilla, R. (2016). Neurotrophin signaling is required for glucose-induced insulin secretion. Dev. Cell *39*, 329–345.

Johannes, G., and Sarnow, P. (1998). Cap-independent polysomal association of natural mRNAs encoding c-myc, BiP, and eIF4G conferred by internal ribosome entry sites. RNA *4*, 1500–1513.

Kuruvilla, R., Ye, H., and Ginty, D.D. (2000). Spatially and functionally distinct roles of the PI3-K effector pathway during NGF signaling in sympathetic neurons. Neuron *27*, 499–512.

Lehigh, K.M., West, K.M., and Ginty, D.D. (2017). Retrogradely transported TrkA endosomes signal locally within dendrites to maintain sympathetic neuron synapses. Cell Rep. *19*, 86–100.

Lianoglou, S., Garg, V., Yang, J.L., Leslie, C.S., and Mayr, C. (2013). Ubiquitously transcribed genes use alternative polyadenylation to achieve tissue-specific expression. Genes Dev. *27*, 2380–2396.

Martin, K.C., and Ephrussi, A. (2009). mRNA localization: gene expression in the spatial dimension. Cell *136*, 719–730.

Mayr, C. (2017). Regulation by 3′-untranslated regions. Annu. Rev. Genet. *51*, 171–194.

Mobley, W.C., Schenker, A., and Shooter, E.M. (1976). Characterization and isolation of proteolytically modified nerve growth factor. Biochemistry *15*, 5543–5552.

Nam, J.W., Choi, S.W., and You, B.H. (2016). Incredible RNA: dual functions of coding and noncoding. Mol. Cells *39*, 367–374.

Nowak, J., Archange, C., Tardivel-Lacombe, J., Pontarotti, P., Pébusque, M.J., Vaccaro, M.I., Velasco, G., Dagorn, J.C., and Iovanna, J.L. (2009). The TP53INP2 protein is required for autophagy in mammalian cells. Mol. Biol. Cell *20*, 870–881.

Raj, A., van den Bogaard, P., Rifkin, S.A., van Oudenaarden, A., and Tyagi, S. (2008). Imaging individual mRNA molecules using multiple singly labeled probes. Nat. Methods *5*, 877–879.

Rappsilber, J., Mann, M., and Ishihama, Y. (2007). Protocol for micro-purification, enrichment, pre-fractionation and storage of peptides for proteomics using StageTips. Nat. Protoc. *2*, 1896–1906.

Riccio, A. (2018). RNA targeting and translation in axons. Science *359*, 1331–1332.

Riccio, A., Pierchala, B.A., Ciarallo, C.L., and Ginty, D.D. (1997). An NGF-TrkA-mediated retrograde signal to transcription factor CREB in sympathetic neurons. Science *277*, 1097–1100.

Sala, D., Ivanova, S., Plana, N., Ribas, V., Duran, J., Bach, D., Turkseven, S., Laville, M., Vidal, H., Karczewska-Kupczewska, M., et al. (2014). Autophagy-regulating TP53INP2 mediates muscle wasting and is repressed in diabetes. J. Clin. Invest. *124*, 1914–1927.

Sambandan, S., Akbalik, G., Kochen, L., Rinne, J., Kahlstatt, J., Glock, C., Tushev, G., Alvarez-Castelao, B., Heckel, A., and Schuman, E.M. (2017). Activity-dependent spatially localized miRNA maturation in neuronal dendrites. Science *355*, 634–637.

Schindelin, J., Arganda-Carreras, I., Frise, E., Kaynig, V., Longair, M., Pietzsch, T., Preibisch, S., Rueden, C., Saalfeld, S., Schmid, B., et al. (2012). Fiji: an open-source platform for biological-image analysis. Nat. Methods *9*, 676–682.

Scott-Solomon, E., and Kuruvilla, R. (2018). Mechanisms of neurotrophin trafficking via Trk receptors. Mol. Cell. Neurosci. *91*, 25–33.

Shigeoka, T., Jung, H., Jung, J., Turner-Bridger, B., Ohk, J., Lin, J.Q., Amieux, P.S., and Holt, C.E. (2016). Dynamic axonal translation in developing and mature visual circuits. Cell *166*, 181–192.

Spivak, M., Weston, J., Bottou, L., Käll, L., and Noble, W.S. (2009). Improvements to the percolator algorithm for Peptide identification from shotgun proteomics data sets. J. Proteome Res. *8*, 3737–3745.

Taverna, D., Mignogna, C., Gabriele, C., Santise, G., Donato, G., Cuda, G., and Gaspari, M. (2017). An optimized procedure for on-tissue localized protein digestion and quantification using hydrogel discs and isobaric mass tags: analysis of cardiac myxoma. Anal. Bioanal. Chem. *409*, 2919–2930.

Terenzio, M., Schiavo, G., and Fainzilber, M. (2017). Compartmentalized signaling in neurons: from cell biology to neuroscience. Neuron *96*, 667–679.

Terenzio, M., Koley, S., Samra, N., Rishal, I., Zhao, Q., Sahoo, P.K., Urisman, A., Marvaldi, L., Oses-Prieto, J.A., Forester, C., et al. (2018). Locally translated mTOR controls axonal local translation in nerve injury. Science *359*, 1416–1421.

Valluy, J., Bicker, S., Aksoy-Aksel, A., Lackinger, M., Sumer, S., Fiore, R., Wüst, T., Seffer, D., Metge, F., Dieterich, C., et al. (2015). A coding-independent function of an alternative Ube3a transcript during neuronal development. Nat. Neurosci. *18*, 666–673.

Xu, Y., Wan, W., Shou, X., Huang, R., You, Z., Shou, Y., Wang, L., Zhou, T., and Liu, W. (2016). TP53INP2/DOR, a mediator of cell autophagy, promotes rDNA transcription via facilitating the assembly of the POLR1/RNA polymerase I preinitiation complex at rDNA promoters. Autophagy *12*, 1118–1128.

Yamashita, N., and Kuruvilla, R. (2016). Neurotrophin signaling endosomes: biogenesis, regulation, and functions. Curr. Opin. Neurobiol. *39*, 139–145.

Yamashita, N., Joshi, R., Zhang, S., Zhang, Z.Y., and Kuruvilla, R. (2017). Phospho-regulation of soma-to-axon transcytosis of neurotrophin receptors. Dev. Cell *42*, 626–639.e5.

Ye, H., Kuruvilla, R., Zweifel, L.S., and Ginty, D.D. (2003). Evidence in support of signaling endosome-based retrograde survival of sympathetic neurons. Neuron *39*, 57–68.

STAR★METHODS

KEY RESOURCES TABLE

REAGENT or RESOURCE	SOURCE	IDENTIFIER
Antibodies		
Goat anti-Tp53inp2 (G-15)	Santa Cruz Biotech	Cat#Sc85972; RRID: AB_2207042
Rabbit anti-Tp53inp2	Sigma-Aldrich	Cat#SAB4502917; RRID: AB_10748208
Rabbit anti-Tp53inp2	This paper	N/A
Rabbit anti-Tp53inp2	Baumgartner et al., 2007	N/A
Mouse anti-Flag M2	Sigma-Aldrich	Cat#F3165; RRID: AB_259529
Mouse anti-Flag	Sigma-Aldrich	Cat#F7425; RRID: AB_439687
Goat anti-HSP90α/β (N-17)	Santa Cruz Biotech	Cat#Sc1055; RRID: AB_2121400
Mouse anti-Myc 4A6	Upstate	Cat#05-724; RRID: AB_309938
Mouse-anti-c-Myc (A7)	Santa Cruz Biotech	Cat#sc56634; RRID: AB_1121739
Mouse anti-c-Myc [9E10]	Abcam	Cat#ab32; RRID: AB_303599
Rabbit anti-P85	Upstate	Cat#06-497; RRID: AB_310141
Rabbit anti-P85	Upstate	Cat#06-195; RRID: AB_310069
Mouse anti-Phosphotyrosine clone PY20	Sigma-Aldrich	Cat#05-947; RRID: AB_477342
Rabbit anti-TrkA	Millipore	Cat#06-574; RRID: AB_310180
Rabbit anti-pAkt	Cell Signaling	Cat#9271; RRID: AB_329825
Mouse anti-pErk1/2	Cell Signaling	Cat#9106; RRID: AB_331768
Mouse anti-α-Tubulin	Sigma-Aldrich	Cat#T9026; RRID: AB_477593
Chicken anti-GFP	Abcam	Cat#Ab13970; RRID: AB_300798
Mouse anti-mCherry	Abcam	Cat#Ab125096; RRID: AB_11133266
Mouse anti-HuD (E-1)	Santa Cruz Biotech	Cat#sc28299; RRID: AB_627765
Mouse anti-Trk (B-3)	Santa Cruz Biotech	Cat#sc7268; RRID: AB_628397
Mouse anti-NCAM-L1 (C-2)	Santa Cruz Biotech	Cat#sc514360
Normal anti-Rabbit IgG	Santa Cruz Biotech	Cat#sc2027; RRID: AB_737197
Normal anti-Mouse IgG	Santa Cruz Biotech	Cat#sc2025; RRID: AB_737182
Sheep Anti-mouse HRP Linked	GE Healthcare life sciences	Cat#NA931; RRID: AB_772210
Donkey Anti-rabbit HRP Linked	GE Healthcare life sciences	Cat#NA934; RRID: AB_772206
Anti-goat HRP Linked	Sigma-Aldrich	Cat#A8919; RRID: AB_258425
Anti-chicken HRP Linked	Sigma-Aldrich	Cat#A9046; RRID: AB_258432
Rabbit anti-NGF	Sigma-Aldrich	Cat#N6655; RRID: AB_477660
Rabbit anti-pTrkA	Cell Signaling	Cat#4168S; RRID: AB_10620952
Mouse anti-TH	Sigma-Aldrich	Cat#T2928; RRID: AB_477569
Rabbit anti-TH	Merck Millipore	Cat#AB152; RRID: AB_390204
Goat Anti-rabbit IgG 488 conjugated	Thermo Fisher	Cat#A11008; RRID: AB_143165
Goat Anti-mouse IgG 647 conjugated	Thermo Fisher	Cat#A21240; RRID: AB_141658
Sheep Alkaline phosphatase-labeled anti-DIG	Roche	Cat# 11093274910; RRID: AB_514497
Mouse anti-β-III-tubulin	Sigma-Aldrich	Cat#T8660; RRID: AB_477590
Bacterial and Virus Strains		
Adenovirus: Cre-expressing	Lois Greene	N/A
Adenovirus: LacZ-expressing	Jeffrey E. Pessin	N/A
Adenovirus: GFP-expressing	Ascano et al., 2009	N/A
Adenovirus: Flag-TrkA expressing	Bodmer et al., 2011; Houtz et al., 2016	N/A

(Continued on next page)

Continued

REAGENT or RESOURCE	SOURCE	IDENTIFIER
Adenovirus: Wildtype *Tp53inp2* expressing	This paper	N/A
Adenovirus: ATGnull *Tp53inp2* expressing	This paper	N/A
Chemicals, Peptides, and Recombinant Proteins		
Nerve Growth Factor	Mobley et al., 1976	N/A
DTAF	Thermo Fisher Scientific	Cat#D16
FluoSpheres	Thermo Fisher Scientific	Cat#F8789
TUNEL	Roche	Cat#11684795910
Cyclohexamide	Sigma	Cat#C7698
EZ-LinkTM NHS-SS-Biotin	Thermo Fisher Scientific	Cat#21441
DAPI	Roche	Cat# 10236276001
Critical Commercial Assays		
AdEasy Adenoviral Vector System	Agilent	Cat#240009
RNAquesous-Micro Total RNA Isolation Kit	Thermo Fisher Scientific	Cat# AM1931
PureLink RNA Micro kit	Thermo Fisher Scientific	Cat#12183016
Lipofectamine 2000 Transfection Reagent	Thermo Fisher Scientific	Cat#11668027
QuikChange Site-Directed Mutagenesis Kit	Agilent	Cat# 210518
Deposited Data		
Raw and analyzed data	This paper	N/A
Experimental Models: Organisms/Strains		
Sprague Dawley rats	UCL biological services unit maintained colony	N/A
Mouse: Tp53inp2fl/fl: 129S4/SvJaeSor-*Gt(ROSA)26Sortm1 (FLP1)Dym/J- Tp53inp2*^{tm1a}	This paper	N/A
Mouse: TH-Cre	Gong et al., 2007	N/A
PC12 cell line	ATCC	Cat# CRL-1721; RRID: CVCL_0481
HEK293 cell line	ATCC	Cat# PTA-4488; RRID: CVCL_0045
HeLa cell line	ATCC	Cat# CRL-7923; RRID: CVCL_0030
PC12 Nnr5 subclone	Green et al., 1986	RRID: CVCL_C128
Oligonucleotides		
Primer for genotyping: Forward Tp53inp2LoxP3F GATCAGGACCTCAGCGATGG	This paper	N/A
Primer for genotyping: Reverse Tp53inp2LoxP3R GCACCTGGCACAGGTAACTA	This paper	N/A
Primers for cloning, see Table S2	This paper	N/A
Primers for qRT-PCR, see Table S2	This paper	N/A
siRNA:ON-TARGET plus Smartpool Rat Tp53inp2 CTAAAGTGTTGCAACGGCA	Dharmacon	J-093056-09
siRNA:ON-TARGET plus Smartpool Rat Tp53inp2 GATCAGGACCTCAGCGATG	Dharmacon	J-093056-10
siRNA:ON-TARGET plus Smartpool Rat Tp53inp2 GATCTAACTCACCTATTAA	Dharmacon	J-093056-11
siRNA:ON-TARGET plus Smartpool Rat Tp53inp2 GACGAGAGCTGGTTTGTTA	Dharmacon	J-093056-12
Recombinant DNA		
Plasmid: mCherry	Clontech	Cat# 632524
Plasmid: pCMV-Myc	Clontech	Cat# 631604
Plasmid: pCMV-mycTp53inp2	This paper	N/A
Plasmid: pcDNA3.1-Tp53inp2CDSNull-2xFlag	This paper	N/A
Plasmid: pcDNA3.1-Tp53inp2CDS-2xFlag	This paper	N/A

(*Continued on next page*)

Continued		
REAGENT or RESOURCE	SOURCE	IDENTIFIER
Plasmid: pCMVShuttle-Wildtype *Tp53inp2*	This paper	N/A
Plasmid: pCMVShuttle-ATGnull *Tp53inp2*	This paper	N/A
Plasmid: pcDNA3.1-5′UTRTp53:myrdEGFP:3′UTRTp1.2	This paper	N/A
Plasmid: pcDNA3.1-5′UTRTp53:myrdEGFP:3′UTRTp2.1	This paper	N/A
Plasmid: pcDNA3.1-5′UTRTp53:myrdEGFP:3′UTRTp3.1	This paper	N/A
Software and Algorithms		
Prism	GraphPad	https://www.graphpad.com/scientific-software/prism/
Openlab 4.0.4	PerkinElmer	
Proteome Discoverer 1.3	Thermo Fisher	https://www.thermofisher.com/order/catalog/product/OPTON-30795
Fiji	Schindelin et al., 2012	https://imagej.net/Fiji

CONTACT FOR REAGENT AND RESOURCE SHARING

Further information and requests for resources and reagents should be directed to and will be fulfilled by the Lead Contact, Antonella Riccio (a.riccio@ucl.ac.uk).

EXPERIMENTAL MODEL AND SUBJECT DETAILS

Generation of Tp53inp2$^{fl/fl}$ mutant mice

ES cells containing the *Tp53inp2*$^{tm1a(KOMP)Mbp}$ targeting vector were obtained from the trans-NIH Knock-Out Mouse Project (KOMP) Repository (https://www.komp.org/) and used by transgenic core facility at Johns Hopkins University to generate chimeric mice carrying the *Tp53inp2*^{tm1a} allele. *Tp53inp2*^{tm1a} chimeric male mice were mated to wild-type albino mice (Jackson Laboratory Stock No: 000058). *Tp53inp2*^{tm1a} mice carry a knockout-first allele, in which a promoterless cassette including LacZ and neo genes were inserted in intron 1 of the *Tp53inp2* gene. For the sympathetic neuron-specific conditional knockout mice, *Tp53inp2*^{tm1a} mice were crossed with a ubiquitously-expressing Flippase line 129S4/SvJaeSor-*Gt(ROSA)26Sortm1(FLP1)Dym/J* (Jackson Lab) to excise the LacZ/neo cassette. These mice were then crossed with *TH-Cre* transgenic mice (gifted by Dr. C. Gerfen NIH) to generate mice lacking *Tp53inp2* in sympathetic neurons. The offspring of these mice were then backcrossed for 2-3 generations to *C57/BL6* mice to obtain offspring of several genotypes including *Th-Cre;Tp53inp2*$^{fl/fl}$ mice and control *Tp53inp2*$^{fl/fl}$ littermates.

Primers *Tp53inp2Loxp3F* and *Tp53inp2Loxp3*R which span the third *loxP* site, were used to genotype the *Tp53inp2* allele by PCR, with expected band sizes being 240 bp for wild-type, and 310 bp for the floxed exon. See Table S2 and Key Resources Table for primer sequences.

Mouse husbandry

Mice were housed in a standard 12:12 light-dark cycle. Both sexes were used for analyses. The ages of mice are indicated in the figure legends or methods. All procedures relating to animal care and treatment conformed to Johns Hopkins University Animal Care and Use Committee (ACUC) and NIH guidelines, or the Institutional Animal Care and Use Committee at University College London.

Primary cultures

Superior cervical ganglia (SCG) were dissected from postnatal day 0 or 1 (P0 or P1) Sprague Dawley rats or *Tp53inp2*$^{fl/fl}$ mice and immediately plated as explant tissue or enzymatically dissociated and cultured either in compartmentalised chambers or as mass cultures. Rats of both sexes were used for analyses. For immunocytochemistry, cells were plated on poly-D-lysine-coated (1 μg/ml) coverslips. Campenot compartmentalised cultures were established as described previously (Riccio et al., 1997). Neurons were cultured in high-glucose DMEM supplemented with 10% FBS, 2 mM glutamine and 100 ng/ml NGF. The antimitotic inhibitor cytosine arabinoside (10 μM) was provided to minimize non-neuronal, contaminating cell types. When indicated, the cell body cluster was removed surgically using a scalpel. To withdraw NGF before any stimulation experiments, neurons were placed in DMEM containing 0.5% FBS with 1:1000 anti-NGF antibody (N6655, Sigma) and BAF (50 μM; MP Biomedical) for 48 hours. For restimulations, neurons were treated with the indicated concentration of NGF for variable amounts of time as indicated.

Mouse cortical neurons

Cortical neurons were dissected from E15.5 C57BL/6J mouse embryos and enzymatically dissociated. Embryos of both sexes were used for analyses. Neurons were plated on Nunc dishes coated with 40 μg/ml poly-D-lysine and 2 μg/ml Laminin and cultured in MEM

supplemented with 10% FBS and 5% Horse serum. After 6-24 h, culture medium was replaced with Neurobasal medium supplemented with B27, 1 mM glutamine, penicillin-streptomycin and 10 μM 5-Fluoro-2′deoxyuridine (FdU, Merck). Cells were cultured at 37°C, 5% CO_2 and one day before the experiment, 2/3 plating medium was replaced with medium without B27 (serum starve conditions).

PC12 cell line

PC12 cell lines were purchased from ATCC. PC12 cells were cultured in DMEM containing 10% FBS, 5% Horse Serum (HS), 2mM glutamine and grown at 37°C 10% CO_2. Cells were transfected with Lipofectamine 2000 in OptiMEM according to the manufacturers' guidelines. For NGF stimulation, cells were cultured in DMEM containing 0.5% FBS, 0.25% HS, 2 mM glutamine and 50 ng/ml NGF for 4 days under standard growth conditions. Cells were not cultured past 20 passages and sensitivity to NGF stimulation routinely tested. The Nnr5 PC12 subclone cell line was a kind gift from David Ginty's Laboratory. Nnr5 cells were cultured in DMEM containing 10% FBS, 5% Horse Serum (HS), 2 mM glutamine and grown at 37°C 10% CO_2. Cells were transfected with Lipofectamine 2000 in OptiMEM according to the manufacturers' guidelines.

HEK293 cell line

The HEK293 cell line was purchased from ATCC and not further authenticated. HEK293 cells were cultured in DMEM containing 5% FBS, penicillin/streptomycin, 2mM glutamine and grown at 37°C 5% CO_2. Cells were transfected with Lipofectamine 2000 in OptiMEM according to the manufacturers' guidelines. Cells were not cultured past 20 passages.

HeLa cell line

The HeLa cell line was purchased from ATCC and not further authenticated. HeLa cells were cultured in MEM containing 5% FBS, penicillin/streptomycin, 2mM glutamine and grown at 37°C 5% CO_2. Cells were transfected with Lipofectamine 2000 in OptiMEM according to the manufacturers' guidelines. For cyclohexamide analysis, cyclohexamide was added directly to the growth media at a final concentration of 20 μg/ml and cells were maintained in culture for times indicated. Cells were not cultured past 20 passages.

METHOD DETAILS

Adenoviral vectors and Plasmids

Rat Tp53inp2CDS was amplified from SCG cDNA and cloned into pCMV-Myc (Clontech) using SalI and BglII to generate Myc-Tp53inp2CDS plasmid. Tp53inp2CDS-2xFlag was PCR amplified from Myc-Tp53inp2CDS plasmid, using primers that encode KpnI/*Xba*I and C terminus 2xFlag tag. Tp53inp2 3′UTR 1.2, 2.2 and 3.1 Kb and 5′UTR were PCR amplified from RACE clones (C.A. and A.R., unpublished data) and following *Not*I/*Xho*I and *BamH*I/*Nhe*I digestion respectively, were used to replace the IMPA UTRs in myrdEGFP-IMPA1-L (Andreassi et al., 2010). Mutation of the ATG codons to generate ATGNullCDS was performed on Myc-Tp53inp2CDS using the QuikChange Site-Directed Mutagenesis kit (Agilent) according to manufacturer's instructions. The ATGNullTp53inp2 CDS was then PCR amplified and used to replace the myrdEGFP sequence in the myrdEGFP-Tp53inp2 3.1 Kb UTR vector to create ATGNull Tp53inp2. Rat Tp53inp2 5′UTR+CDS was amplified from SCG cDNA to include endogenous Kozack sequence, and cloned in place of the ATGNull CDS to generate WT Tp53inp2 vector. To generate adenoviral vectors, WT Tp53inp2 and ATGNull Tp53inp2 were amplified using primers containing KpnI and *Eco*RV and inserted into pCMV-Shuttle plasmid (Agilent). Adenoviral vectors were generated using the AdEasy Adenoviral Vector system (Agilent) according to manufacturer's instructions. Adenoviral vectors were transfected into HEK293 cells and high-titer viral stocks were prepared using a CsCl gradient. Tp53inp2CDS-1xFlag and ATGNullCDS-1xFlag were PCR amplified from Myc-Tp53inp2CDS and ATGNullCDS Tp53inp2 respectively and following BamHI/*Not*I digestion were cloned into pcDNA3.1. Primers used for cloning are listed in Table S2.

RNA isolation and RT-qPCR

Samples were washed with PBS and RNA was extracted using Trizol reagent following standard manufacturer's protocol. RNA pellet was resuspended in water and contaminating genomic and plasmid DNA removed by DNase digestion with TURBO DNase. PCR was performed to amplify Actin gDNA or plasmid DNA prior to reverse transcription to confirm DNase digestion. For cell bodies excised from explants of sympathetic neurons used in the axonal TrkA signaling assays, RNA was isolated using RNAquesous-Micro Total RNA Isolation Kit (ThermoFisher) per manufacturer's protocol with recommended DNase treatment. Total RNA was reverse transcribed using random hexamers and SuperscriptIII reverse transcriptase. RT-qPCR was performed using 20 μL reactions using MESA Blue (Eurogentec), SYBR green or SYBR Select master mix in the Mastercycler realplex qPCR machine (Eppendorf) or CFX Connect RT-PCR machine (BIORAD). Reactions were performed in triplicate and a no template control. For absolute quantification, a standard curve was included, generated through serial dilutions of known concentration of the DNA amplicon for each primer set. For relative quantification, the power(2,-Ct) of the experimental gene was normalized by the power(2,-Ct) of a housekeeping gene and then expressed as fold change relative to a control. Following 40 cycles, a dissociation curve was performed to assess melting temperature of amplicons. The primers used for RT-qPCR in this study are listed in Table S2.

In situ hybridization

In situ hybridization was performed using a digoxigenin-labeled probe spanning a 450-bp region within exons 2-3 of mouse Tp53inp2. Embryos or torsos at various developmental stages were fresh frozen in OCT and serially sectioned (12 μm) using a cryostat. Tissue sections from different developmental stages were collected and processed simultaneously. Sections were post-fixed in 4% PFA-PBS, washed in PBS and acetylated with 0.25% acetic anhydride in 0.1 M triethanolamine with 0.9% NaCl. After hybridization with the labeled RNA probe (2 μg/ml) at 68 °C o/n, sections were washed with 0.2x SSC buffer at 65 °C, blocked with TBS containing 1% normal goat serum and then incubated with alkaline phosphatase-labeled anti-DIG antibody (1:5,000; Roche) o/n at 4 °C. The alkaline phosphatase reaction was visualized with NBT/BCIP, rinsed in PBS, fixed in 4% PFA-PBS and mounted in AquaMount (EMD Chemicals).

smFISH

smFISH was performed as described (Chen et al., 2015; Raj et al., 2008) with minor modifications. Sympathetic neurons were cultured 3-7 days *in vitro* on glass coverslips, washed with PBS at room temperature (RT) and fixed using 3.7% PFA-PBS RT 10 min. Cells were permeabilised with 70% EtOH at 4°C for 3 h. Coverslips were prehybridized in 2xSSC 10% formamide for 5 min RT then incubated at 37°C o/n with Cy3 labeled probe sets designed against *Tp53inp2* 3′UTR (Stellaris probes, Biosearch technologies) in hybridization buffer (10% formamide, 2x SSC, 10% dextran sulfate and 2 mM vanadyl ribonucleoside). Cells were rinsed in warm 2x SSC 10% formamide and then 5-(4,6-dichlorotriazinyl) aminofluorescein (DTAF) (Thermo Fischer, D16), a reactive dye that labels amines in proteins, was added at 1:10,000 (stock 10 mg/ml) in GLOX buffer for 10 min as a counterstain. Cells were then rinsed in 2x SSC and mounted using Aquamount containing 1:1000 DAPI.

Neuronal counts

The torsos of *Tp53inp2*$^{fl/fl}$ or *Th-Cre;Tp53inp2*$^{fl/fl}$ E16.5, P0.5 and P21 mice were immersion fixed in 4% PFA-PBS for 4 h (E16.5, P0.5), or overnight (P21) respectively. The heads were then cryoprotected overnight (for E16.5 and P0.5) or 2 days (P21) in 30% sucrose-PBS, frozen in OCT and then serially sectioned (12 μm). Tissue sections were stained with a solution containing 0.5% cresyl violet (Nissl). Cells with characteristic neuronal morphology and visible nucleoli were counted in every fifth Nissl-stained section. For representative images, immunohistochemistry was performed on tissue sections. Sections were first blocked in a PBS buffer containing 5% goat serum and 0.1% triton and then incubated overnight in rabbit anti-TH (1:200; Millipore AB152). Following PBS washes; sections were incubated with anti-rabbit Alexa 488 secondary antibodies (1:200; Life Technologies). Sections were then washed in PBS and mounted in VectaShield (Vector Laboratories). For practical reasons, analyses were done in a semi-blinded manner, with the investigator knowing the genotypes prior to the experiment, yet performed the staining and quantification without knowing the genotype of the sample.

Whole-mount diaminobenzidine-TH immunohistochemistry

E16.5 embryonic organs fixed in 4% PFA-PBS were dehydrated by methanol series (50%–80%) and incubated overnight in 20% dimethylsulfoxide (DMSO)/80% methanol solution containing 3% H_2O_2 to quench endogenous peroxidase activity. Tissues were then re-hydrated, blocked o/n in blocking solution (4% BSA/1% Triton X-100 in PBS) using a rabbit anti-TH (Millipore, AB152) diluted at 1:200 in blocking solution and incubated for 72 h at 4°C. Detection was performed with horseradish peroxidase-conjugated donkey anti-rabbit IgG (GE Healthcare) at 1:200 in blocking solution and incubated o/n at 4°C. Visualization was accomplished with diaminobenzidine. Tissues were re-fixed in 4% paraformaldehyde-PBS, dehydrated by methanol series and cleared with a 2:1 mixture of benzyl benzoate/benzyl alcohol to allow visualization of staining inside the tissue followed by clearing. For practical reasons, analyses were done in a semi-blinded manner, with the investigator knowing the genotypes prior to the experiment, yet performed the staining and quantification without knowing the genotype of the sample.

Neuronal survival

SCG neurons isolated from P0.5-P2 *Tp53inp2*$^{fl/fl}$ were grown in Campenot chambers for 9 days *in vitro*. NGF was removed and neurons were infected for 48-72 h with either LacZ or Cre adenovirus. FluoSpheres (ThermoFisher: F8789) were added to axonal compartments to label projecting neurons. Neurons were starved of NGF and restimulated with 25 ng/ml NGF for 48 h or left deprived. To detect neuronal death, neurons were fixed in 4% PFA-PBS for 30 min at room temperature followed by permeabilisation with 0.1% Triton/PBS for 10 min. After extensive PBS washes, dying neurons were visualized using TUNEL staining (Roche: 11684795910) and mounted in Fluoromount with DAPI (1:1000). Neuronal apoptosis was calculated by determining the percentage of neurons that had extended to distal compartments that were also TUNEL positive. Between 30-50 neurons were counted per condition in each experiment.

Axon growth

Compartmentalised neuronal cultures from P0.5 *Tp53inp2*$^{fl/fl}$ neurons were infected with LacZ or Cre adenovirus after 7-10 days *in vitro* after axons had extended into the side compartments. Neurons were either deprived or treated with NGF (100 ng/ml) added solely to the axonal compartments, and axon growth was measured by capturing phase contrast images of the distal axons over consecutive 24 h intervals for 3 days, using a Zeiss Axiovert 200 microscope with a Retiga EXi camera. The rate of axonal growth

(μm/day) was quantified using Openlab 4.04. For representative images, neurons were immunostained with β-III-tubulin (1:200; Sigma-Aldrich T8660) 48 h after treatment.

Polysomal Fractionation

SCG neurons were cultured for 7 days under NGF stimulation, then ribosomes were immobilised with 0.1 mg/ml cyclohexamide added directly to growth media for 3 min at 37°C. Cells were washed 2x on ice with cold PBS + 0.1 mg/ml cyclohexamide and lysed in 10 mM Tris pH8, 150 mM NaCl, 5 mM MgCl2, 1% NP40, 10 mM VRC, 1% sodium deoxycholate, 40 mM DTT, 500 U/ml RNaseOUT. Nuclei were removed by a brief centrifugation and supernatants were supplemented with 0.1 M Tris pH7.5, 150 mM NaCl, 75 μg/ml cyclohexamide, 1:100 protease inhibitor cocktail, followed by further centrifugation to remove any insoluble material. Lysates were loaded on top of 15%–40% sucrose gradients, prepared in 10 mM Tris pH7.5, 140 mM NaCl, 1.5 mM $MgCl_2$, 10 mM DTT, 0.1 mg/ml cyclohexamide, and subjected to ultracentrifugation in Beckman SW41Ti rotor 2 h 4°C 25000x*g*. Nineteen fractions were collected and digested with proteinase K (100 μg proteinase K, 0.1% SDS, 10 mM EDTA) for 30 min at 37°C. To normalize for RNA loss during extraction, 2.5 ng of *in vitro* transcribed RNA was spiked into each fraction prior to phenol:chloroform extraction. Twenty-five per cent of sample was precipitated for RT-qPCR, and the remainder was precipitated for denaturing agarose gel electrophoresis to assess quality of the fractionation. RNA was run on 1% agarose MOPS gel under denaturing conditions and the gel was stained with SYBRGold prior to imaging.

Mass Spectrometry

PC12 cells transfected with a plasmid expressing Myc-Tp53inp2 were washed with cold PBS and lysed in cold RIPA buffer (50 mM Tris pH 7.4, 150 mM NaCl, 0.5% sodium deoxycholate, 1% NP40, 1:100 Protease inhibitor cocktail). Lysates were sheared through a 25 G needle and protein content was quantified by BCA assay according to manufacturer's instructions (Pierce). 2 μg Myc 9E10 antibody (Ab32, Abcam) was conjugated to protein G-Sepharose (GE Healthcare Life Science) o/n rotating at 4°C. Bound antibody was then cross-linked to the beads with 2x30 min RT rotation with 6.5 mg/ml DMP (Thermo Fisher Scientific) in 0.2 M pH8.2 triethanolamine, followed by 30 min RT blocking in 100 mM pH8.2 ethanolamine. Beads were washed extensively with PBS and the unbound antibody eluted with 0.1 M Glycine 10 min RT. Following further PBS washes of beads, 300 μg of protein lysate was added and incubated o/n rotating at 4°C. Beads were washed 3 times 10 min RT in wash buffer (10 mM Tris pH7.4, 150 mM NaCl, 1 mM EDTA pH8, 1 mM EGTA pH8, 0.1% Triton X-100) followed by a final PBS wash. Samples were then subjected to on-bead digestion using 200 ng sequencing grade trypsin in 300 μL 100 mM Tris pH8 15 min 37°C. Beads were pelleted by centrifugation and supernatant collected and analyzed by mass-spectrometry. Lyophilised samples were reconstituted in HPLC-grade water and reduced with 10 mM DTT 1h at 37°C, followed by cysteine alkylation (25 mM iodoacetamide, 1 h at 37°C in the dark); excess iodoacetamide was quenched by 1 μL of 100 mM DTT. Full tryptic digestion was achieved by an additional overnight incubation in presence of 200 ng of trypsin proteomics grade. Peptides were purified by strong cation exchange (SCX) extraction tips (Rappsilber et al., 2007), and eluted in 7 μL of 500 mM ammonium acetate containing 20% acetonitrile v/v. A 10% aliquot of the IP digest was injected for nanoLC-MS/MS analysis in data-dependent mode.

SCG explants were cultured under NGF stimulation for 7 days and axons surgically dissected from cell body. Axon and cell body samples were then lysed in cold RIPA buffer, quantified by BCA and analyzed by mass-spectrometry. Proteins were precipitated by adding four volumes of cold acetone, and by incubating the solution at −20°C for 1 h. After a centrifugation step carried out at 12,000x*g* for 20 min 4°C, the supernatant was discarded, and the pellet was resuspended in buffer containing 8 M urea, 100 mM Tris pH 8.5 and 0.2% w/v SDS. Protein reduction and alkylation was performed as described above. The protein solution was brought to a final volume of 400 μL by adding HPLC-grade water to reduce urea and SDS concentrations. Finally, 2 μg of trypsin proteomics grade was added to each sample and incubated o/n at 37°C with agitation. The digests were diluted 8-fold in 80% acetonitrile/0.5% formic acid before being loaded onto SCX extraction tips. For targeted analysis, 30 μL of the initial digest solutions were loaded on SCX extraction tips and then eluted in 7 μL of 500 mM ammonium acetate containing 20% acetonitrile (v/v); the eluate was evaporated to dryness and reconstituted in 20 μL of mobile phase A (0.1% formic acid, 2% acetonitrile); a 3 μL aliquot was then injected for nanoscale liquid chromatography coupled to tandem mass spectrometry (nanoLC-MS/MS) analysis.

NanoLC-MS/MS analysis

Proteomic analysis by nanoLC-MS/MS was essentially performed as described in Taverna et al. (2017). Briefly, peptides were loaded directly on-column at 500 nL/min and separated at 300 nL/min via a 60-min gradient ramped from 5% to 35% mobile phase B (80% acetonitrile, 0.1% formic acid); mobile phase A was 2% acetonitrile, 0.1% formic acid. The analytical column, a pulled 75 μm i.d capillary in-house packed to a length of 12 cm, was connected to a liquid chromatography system (EasyLC 1000, Thermo Fisher Scientific) and directly interfaced to a quadrupole-orbitrap mass spectrometer Q-Exactive (Thermo Fisher Scientific) via a nanoelectrospray interface operating in positive ion mode. Data-dependent acquisition was performed using a top-12 method. Full scan parameters were: resolution 70,000, m/z range 350-1800. Tandem mass spectrometry parameters were: resolution 17,500, isolation window 1.6 m/z, maximum injection time 60 ms, AGC target 100,000, normalized collision energy 25, dynamic exclusion 30 s. Data were processed using Proteome Discoverer 1.3 (Thermo Fisher Scientific). Tandem mass spectrometry data were searched against the Rattus norvegicus Uniprot database (downloaded on 03/2015) merged with a list of common contaminating proteins (albumin, trypsin, human keratins). Sequest search parameters were: MS tolerance 20 ppm; MS/MS tolerance 0.02 Da; oxidised

methionine (variable); carbamidomethyl cysteine (static); enzyme trypsin; maximum missed cleavages 2. Search results were filtered by Percolator (Spivak et al., 2009), integrated in Proteome Discoverer, using default parameters. Protein hits based on two high-confidence peptide identifications (q-value < 0.01) were considered valid.

For targeted mode of analysis, the Orbitrap analyzer was operated in targeted MS/MS mode: a single full MS scan (17,500 resolution, 500,000 target ions) was followed by three targeted MS/MS events on precursors at 420.2 (ALHHAAAPMoxPAR, z = 3), 507.6 (HQGSFIYQPCQR, z = 3), 414.9 (ALHHAAAPMPAR, z = 3) m/z. MS/MS conditions were, in all cases, the following: MS resolution 70,000, maximum injection time 250 ms, isolation window 2.0 m/z, AGC target 200,000, normalized collision energy 25.

RNA Immunoprecipitation

Four μg Trk antibody (Santa Cruz sc7268), HuD antibody (Santa Cruz sc28299), NCAM-L1 antibody (Santa Cruz sc514360) or Mouse IgG antibody (Santa Cruz sc2027) were incubated with prewashed protein A/G agarose beads (Santa Cruz) in a PBS buffer containing 1 mg/ml heparin and 1% BSA for 2 h 4°C, and then washed with wash buffer (50 mM Tris pH8, 150 mM NaCl, 1% Triton X-100). Rat SCG neurons or mouse cortical neurons were cultured 5-7 days *in vitro* and then, for SCG cultures, deprived of NGF for 24 h before stimulating for 30 min with NGF or leaving unstimulated. Cultures were washed with PBS and lysed in Buffer A (50 mM Tris pH8, 150 mM NaCl, 1% Triton X-100, 1:100 Protease inhibitor cocktail, 500 U/ml RNaseOUT) 10 min 4°C. Insoluble material was removed by centrifugation, a 10% fraction saved for total input, and lysates were incubated with antibody conjugated beads for 1 h at 4°C in wash buffer containing 0.2 mg/ml heparin. Beads were washed 3 times with wash buffer 10 min 4°C followed by elution of RNA in extraction buffer (0.2 M NaAcetate, 1 mM EDTA, 0.2% SDS) for 5 min at 70°C. RNA was purified from inputs and immunocomplexes using PureLink® RNA Micro Scale Kit according to manufacturer's instructions and analyzed by qRT-PCR.

Immunoprecipitation and immunoblotting analyses

SCGs were isolated from P0-P3 *Tp53inp2*$^{fl/fl}$ mice and grown as explant cultures. After 7-8 days *in vitro*, NGF was removed and neurons infected for 48 h with either LacZ or Cre adenovirus. Cell bodies were surgically removed and the axons were stimulated with 50 ng/ml NGF for 30 min or treated with anti-NGF antibody. Three to seven explants per condition were lysed in RIPA buffer with 2.5 mM sodium pyrophosphate, 50 mM sodium fluoride, 1 mM sodium orthovanadate and protease inhibitors. To detect P-TrkA, axon lysates were subjected to immunoprecipitation with anti-phosphotyrosine antibody (PY-20; 4 μl; Sigma-Aldrich) and incubated with Protein G-Plus (Santa Cruz: sc-2002) agarose beads. Immunoprecipitates were then immunoblotted for TrkA (1:1000 Millipore; 06-574). The supernatants from the immunoprecipitations were subjected to immunoblotting with pAkt (1:1000 Cell Signaling; 9271), pErk1/2 (1:1000 Cell signaling; 9106) and p85 antibodies (1:1000 Upstate Biotechnology, 06-195). All immunoblots were visualized with ECL Plus Detection Reagent (GE Healthcare) and scanned with a Typhoon 9410 Variable Mode Imager (GE Healthcare).

For Trk and HuD immunoprecipitations, PC12 cells or SCG neurons were lysed in RIPA buffer (50 mM Tris-HCl pH 7.4, 150 mM NaCl, 1% NP-40, 0.5% Sodium deoxycholate, 0.1% SDS, 1 mM EDTA, Protease Inhibitors Cocktail) for 10 min on ice, and then insoluble material was removed by centrifugation. Lysates were pre-cleared and 0.5-1 mg of lysate was incubated o/n at 4°C with 2 μg of indicated antibody. Immuno-complexes were precipitated by the addition of protein A-agarose beads at 4°C for 2 h, and then beads washed extensively with RIPA buffer. Samples were eluted from beads by boiling at 95°C in 1x LDS buffer + 10% b-mercaptoethanol and resolved on polyacrylamide gels.

In all other western blots, cell lysates were prepared in 1x NuPAGE LDS buffer + 10% β-mercaptoethanol, mechanically disrupted by syringing and boiled at 95°C for 5 min before loading. The primary antibodies used were goat anti-Tp53inp2 (1:250 Santa Cruz sc85972), rabbit anti-Tp53inp2 (1:200 generated by Riccio Laboratory) rabbit anti-Tp53inp2 (1:500 a kind gift from Zorzano Laboratory; Baumgartner et al., 2007), rabbit anti-Tp53inp2 (1:1000 Sigma AB4502917) mouse anti-Flag (1:1000 Sigma F3165), rabbit anti-Flag (1:1000 Sigma F7425), goat anti-Hsp90 (1:1000 Santa Cruz sc1055), mouse anti-Myc (1:2000 Upstate 05-724), rabbit anti-p85 PI3K (1:1000 Upstate 06-497), mouse anti-α-Tubulin (1:10000 Sigma T9026), chicken anti-GFP (1:5000 Abcam ab13970) mouse anti-mCherry (1:4000 Abcam ab125096), mouse anti-HuD (1:1000 Santa Cruz sc28299), mouse anti-Trk (1:1000 Santa Cruz sc7268) mouse anti-NCAM (1:1000 Santa cruz sc514360). The HRP-conjugated secondary antibodies used were anti-mouse (GE Healthcare life sciences) anti-rabbit (GE Healthcare life sciences), anti-goat (Sigma), anti-chicken (Sigma). Signal was detected using ECL or ECL Prime (GE Healthcare Life Science) and exposing membrane to Amersham Hyperfilm (GE Healthcare Life Science).

pTrkA Immunostaining

SCG neurons isolated from P0-P3 *Tp53inp2*$^{fl/fl}$ mice were grown in Campenot chambers for 8-10 days *in vitro*. NGF was removed and neurons infected for 48 h with either LacZ or Cre adenovirus. FluoSpheres were added to axonal compartment to label projected neurons. Distal axons were treated for 4 h with either 50 ng/ml NGF or anti-NGF antibody. To detect pTrkA in the cell bodies induced by retrograde NGF signal, neurons were fixed in 4%PFA-PBS for 30 min at room temperature and blocked in a PBS buffer containing 5% Goat Serum and 0.1% TritonX for 1 h. Neurons were incubated with rabbit anti-pTrkA (1:1000; Cell Signaling Technology, 4168S) and mouse anti-TH antibodies (1:1000, Sigma, T2928) in a PBS buffer containing 1% Goat Serum and 0.1% Triton, o/n at 4°C. Following extensive washes with PBS, neurons were incubated with fluorescently conjugated anti-rabbit (ThermoFisher, A11008) and anti-mouse antibodies (Thermo Fisher, A21240) secondary antibodies at 1:1000 dilution, 37°C for 2 h. Slides were mounted in Fluoromount (Sigma) with DAPI. Images representing 0.8 μm slices were acquired using a Zeiss LSM 700 confocal scanning microscope. The same confocal settings were used to acquire all images taken from a single experiment. For practical reasons,

analyses were done in a semi-blinded manner, with the investigator knowing the genotypes prior to the experiment, yet performed the staining and quantification without knowing the genotype of the sample.Total pTrkA positive punctae were quantified using Particle Analyses for 20-30 neurons per condition.

Analyses of surface TrkA, receptor endocytosis, and total TrkA

Live-cell antibody feeding to monitor surface TrkA and receptor endocytosis were performed as previously described (Yamashita et al., 2017). Cultured sympathetic neurons isolated from *Tp53inp2*$^{fl/fl}$ mice were infected o/n with a doxycycline-inducible Flag-TrkA adenovirus, and either LacZ or Cre adenovirus. Neurons were then treated with doxycycline (200 ng/mL, 18 hr) to induce Flag-TrkA expression. Surface Flag-TrkA was labeled by incubating neurons with anti-Flag antibody (Sigma, F7425, 1:500) in PBS for 30 mins at 4°C, in the absence of NGF. Excess antibody was washed off with ice-cold PBS, and neurons either immediately fixed with 4% PFA/PBS for 30 mins to assess surface Flag-TrkA distribution, or treated with anti-NGF or NGF (50 ng/mL) for another 30 min to assess receptor internalization. Following treatment with NGF or anti-NGF, neurons were returned to 4°C and quickly washed in ice-cold acidic buffer (0.2 M acetic acid, 0.5 M NaCl, pH 3.0) to strip surface-bound Flag antibodies and fixed. Neurons were permeabilised with 0.1% Triton X-100/5%Normal Goat Serum/PBS and receptors visualized by incubation with anti-rabbit-Alexa 488 secondary antibody. Neuronal morphology was visualized by co-staining with anti-β-tubulin III antibody (Sigma-Aldrich, T8660, 1:1000). Images representing 0.8 μm slices were acquired using a Zeiss LSM 700 confocal scanning microscope. The same confocal settings were used to acquire all images taken from a single experiment. For practical reasons, analyses were done in a semi-blinded manner, with the investigator knowing the genotypes prior to the experiment, yet performed the staining and quantification without knowing the genotype of the sample. Surface Flag-TrkA receptor distribution was analyzed by measuring the integrated fluorescence values along the longest axis of the cell body using line-plot in ImageJ and normalized to the total cell body fluorescence intensity. Intracellular accumulation of Flag-TrkA receptors in cell bodies were quantified as the number of FLAG-immunopositive punctae per neuron. Cell bodies were visualized using the GFP signal and FLAG signals overlapping with GFP fluorescence were defined as internalized soma surface-derived receptors. To assess Flag-TrkA internalization, cell bodies or axons were outlined using β-tubulin III immunostaining and internalized receptors calculated as integrated density of Alexa 488 pixels per μm2 (cell bodies) or Alexa 488 pixels per μm (axons).

Cell surface biotinylation was performed on *Tp53inp2*$^{fl/fl}$ neurons as previously described (Houtz et al., 2016). Briefly, *Tp53inp2*$^{fl/fl}$ neurons, infected with either LacZ or Cre adenoviruses, were deprived of NGF in the media after a culture period of 8-10 d.i.v. and biotinylated at 4°C with a reversible membrane-impermeable form of biotin (EZ-Link NHS-SS-Biotin, 1.5 mg/ml in PBS, ThermoFisher Scientific, Cat# 21441) for 30 min. Cells were washed briefly with PBS containing 50 mM glycine (Sigma) to remove remaining unconjugated biotin, and then moved to 37°C and stimulated with NGF (50 ng/mL) for 30 min to promote internalization. Cells were returned to 4°C, the remaining biotinylated surface receptors were stripped of their biotin tag with 50 mM glutathione (Sigma), followed by two washes with 50 mM iodoacetamide (Sigma) to quench excess glutathione. Cells were lysed with 500 μl of RIPA buffer (50mM Tris-HCl, 150 mM NaCl, 1 mM EDTA, 1% NP-40, 0.25% deoxycholate), and supernatants subjected to precipitation with 40 μl-immobilized neutravidin agarose beads (ThermoFisher Scientific) and immunoblotted for TrkA.

QUANTIFICATION AND STATISTICAL ANALYSIS

Data are expressed as average ± SEM. One-way ANOVA, two-way ANOVA, Mann-Whitney two-tailed test, or t test were used as indicated in the figure legends to test for statistical significance using GraphPad Prism. *n* values are indicated in the corresponding figure legends. Significance was placed at $p < 0.05$ unless otherwise noted in the figure legends. Statistical methods were not used to determine sample size, but sample size was selected based on similar studies within the field.

DATA AND SOFTWARE AVAILABILITY

The datasets generated during the current study are available from the corresponding author on reasonable request.

Neuron, Volume *102*

Supplemental Information

Regulation of NGF Signaling by an Axonal Untranslated mRNA

Hamish Crerar, Emily Scott-Solomon, Chantal Bodkin-Clarke, Catia Andreassi, Maria Hazbon, Emilie Logie, Marifé Cano-Jaimez, Marco Gaspari, Rejji Kuruvilla, and Antonella Riccio

Figure S1

A

GeneID	GeneSymbol	**Axon** Log2 Read Count (Average)	**Cell Body** Log2 Read Count (Average)	Axon:Cell Body Log2 Ratio
ENRNOT00000055310	Tp53inp2	18.150	9.091	1.997
ENRNOT00000015034	Trak2-202	15.205	6.627	2.294
ENRNOT00000014939	Trak2-201	15.204	6.627	2.294
ENRNOT00000030919	Fth1	14.888	9.292	1.602
ENRNOT00000071069	Nefm	13.547	12.422	1.091
ENRNOT00000027691	Apc	13.338	7.882	1.692
ENRNOT00000001383	Tpt1	13.296	8.176	1.626
ENRNOT00000004921	Sytl4-202	13.292	5.383	2.469
ENRNOT00000004909	Sytl4-201	13.292	5.382	2.470
ENRNOT00000028315	Ftl1	13.276	8.248	1.610

B

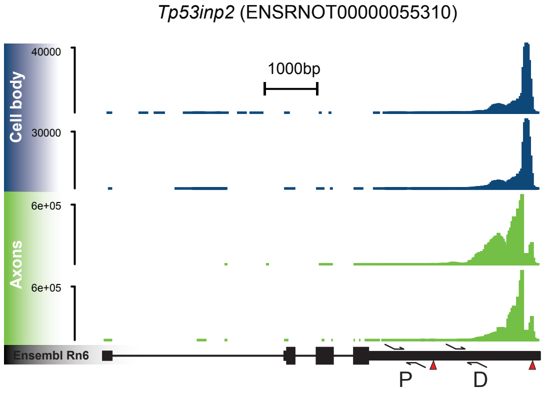

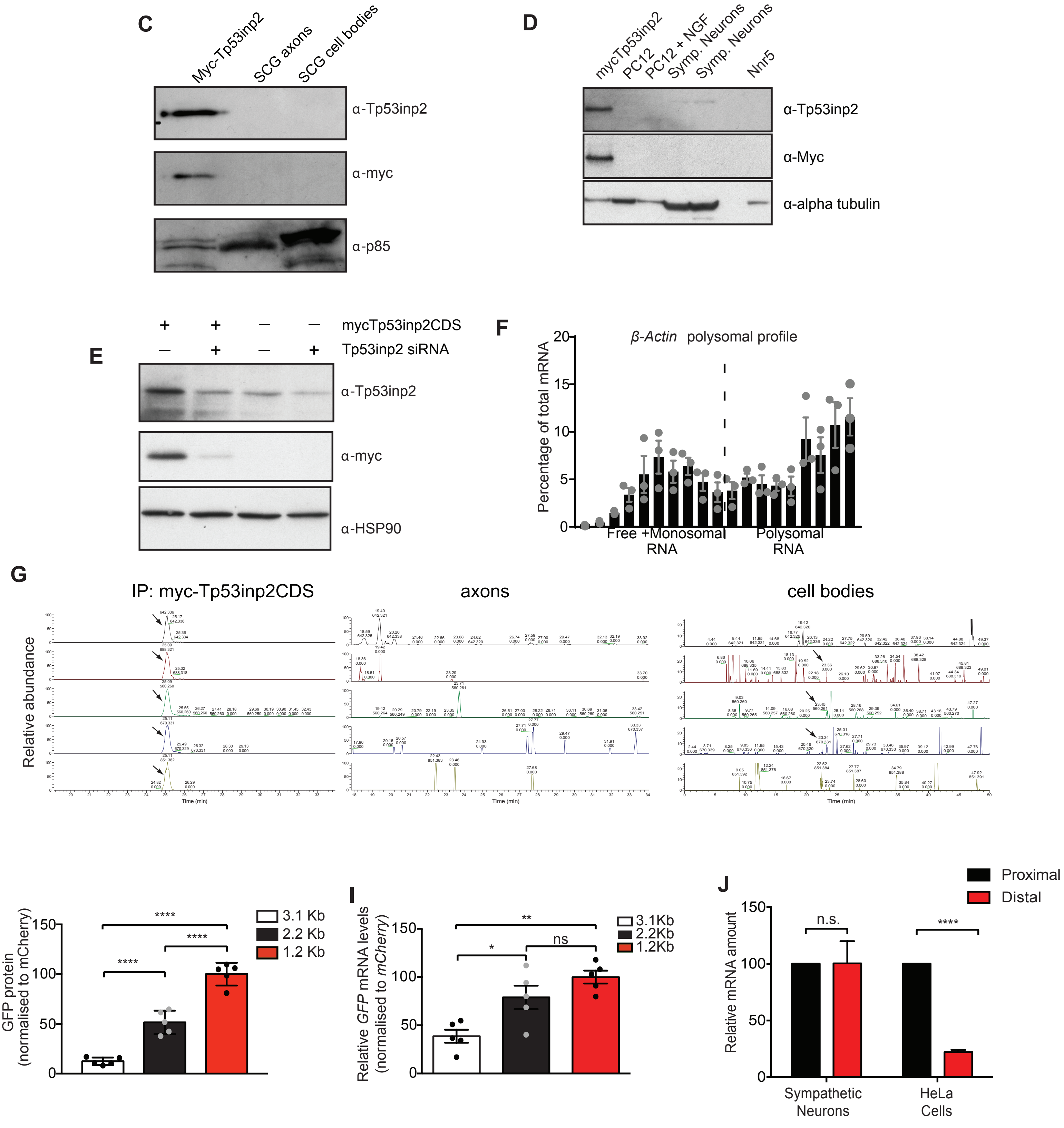

Figure S1: *Tp53inp2* translation is repressed in sympathetic neurons. Related to Figure 1. A, Top ten most abundant axonal transcripts as identified by 3'end RNAseq screen performed on axons and cell bodies of sympathetic neurons. **B**, (*upper*) 3'end Seq tracks of *Tp53inp2* mRNA from cell body and axonal mRNA and (l*ower*) schematic of Ensembl transcript ID ENSRNOT00000055310, red arrowhead represent the positions of AATAAA PAS hexamer, black arrows represent the position of proximal (P) and distal (D) primers used for analysis in **Figure S1J**. **C**, Western blotting analysis of Tp53inp2, Myc and PI3K subunit of p85 on PC12 lysates transfected with Myc-Tp53inp2 CDS, and axonal and cell body lysates of sympathetic neurons. See also (**Figure 1A,B**). **D**, Western blot analysis of PC12 cells transfected with mycTp53inp2, PC12 cells ±NGF stimulation, SCG sympathetic neurons and Nnr5 PC12 cell line. **E**, Western blot analysis of HeLa lysates co-transfected with mycTp53inp2CDS and Tp53inp2 siRNA, as indicated (*n*=3). (See also **Figure 1B**). **F,** Polysomal fractionation profile of *β-actin* mRNA from sympathetic neurons. Values are expressed as percentage of total *β-actin* mRNA (*n*=3). (See also **Figure 1C**). **G,** *Pseudo*-Selected Reaction Monitoring traces for the detection of a Tp53inp2 tryptic peptide in cultured sympathetic neuron axon or cell body samples, and in immunoprecipitated mycTp53inp2 control. The 5 traces represent the 5 most abundant fragments of the Tp53inp2 peptide HQGSFIYQPCQR (*m/z* 507.6). Arrows indicate where at least three transitions are detected at the same retention time. Top value on trace=retention value, bottom value=m/z. (See also **Figure 1D-F**). **H**, Densitometry of GFP protein levels normalised by mCherry levels and expressed as percentage of the mean GFP protein amount of the 1.2 Kb construct. Data presented as average ±s.e.m.; Ordinary one-way ANOVA Tukey's multiple comparisons (*n*=5, ****P<0.0001). See also (**Figure 1G**). **I**, Relative *GFP* mRNA expression in PC12 cells transfected with GFP constructs carrying *Tp53inp2* 3'UTR of indicated length (Kb). mRNA levels were normalised to levels of *mCherry* mRNA and expressed as percentage of the mean *GFP* amount of the 1.2 Kb construct. Data are presented as

averages ±s.e.m., ordinary one-way Tukey's multiple comparisons ANOVA (*n*=5, *P<0.05 **P<0.01 n.s. = not statistically significant). (See also **Figure 1G**). **J,** RT-qPCR analysis of *Tp53inp2* mRNA expression in rat sympathetic neurons and HeLa cell lysate. Primers proximal and distal of the internal polyA site in rat *Tp53inp2* 3'UTR (see **Figure S1B**) were used for analysis and values are normalised to percentage expression of the respective proximal primer set. Data are presented as average ±s.e.m. multiple t-test (*n*=6, ****P<0.0001 n.s. = not statistically significant).

Figure S2

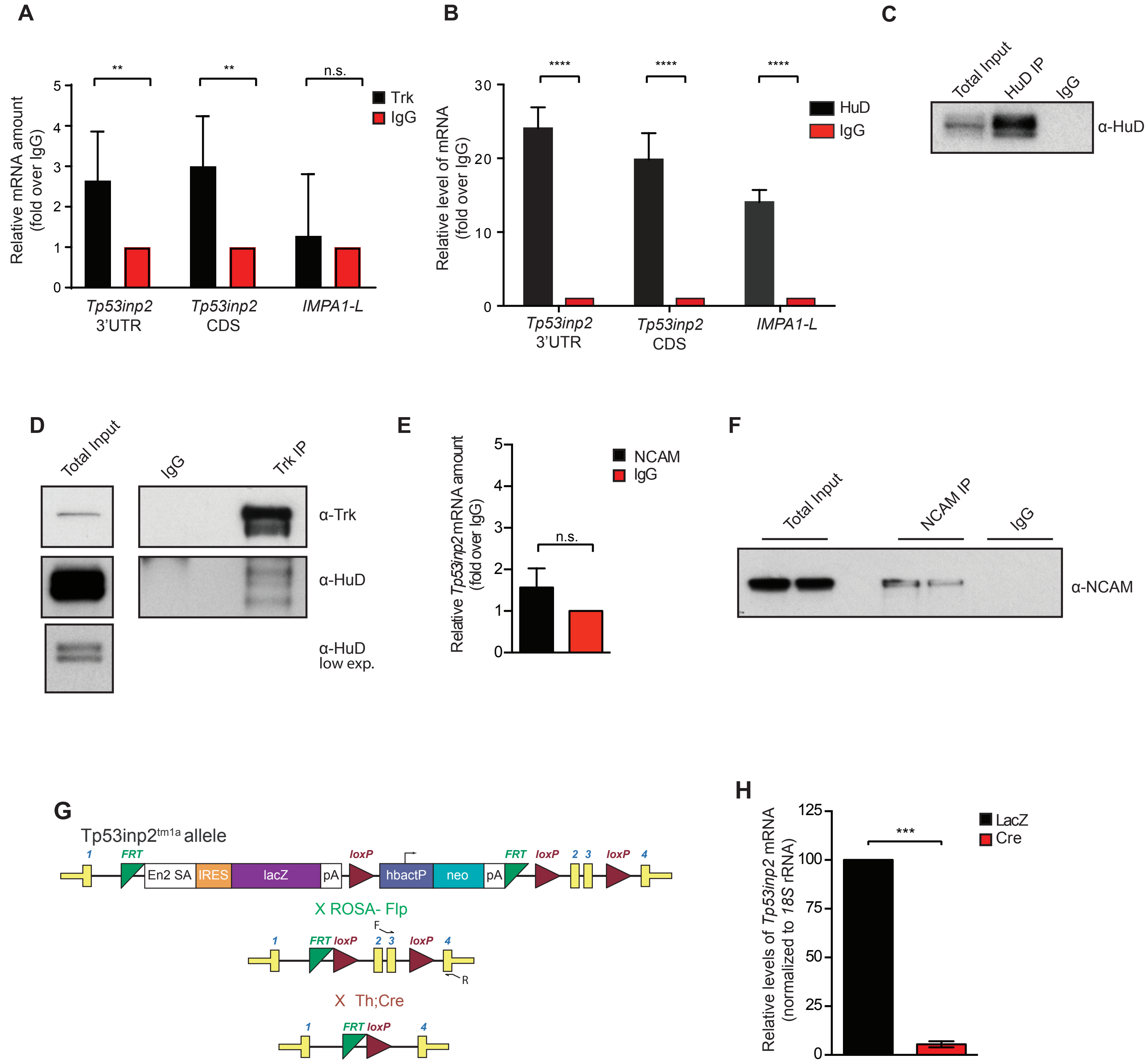

A
Relative mRNA amount (fold over IgG)
**
**
n.s.
Trk
IgG
Tp53inp2 3'UTR
Tp53inp2 CDS
IMPA1-L
B
Relative level of mRNA (fold over IgG)
****
****
****
HuD
IgG
Tp53inp2 3'UTR
Tp53inp2 CDS
IMPA1-L
C
Total Input
HuD IP
IgG
α-HuD
D
Total Input
IgG
Trk IP
α-Trk
α-HuD
α-HuD low exp.
E
Relative Tp53inp2 mRNA amount (fold over IgG)
n.s.
NCAM
IgG
F
Total Input
NCAM IP
IgG
α-NCAM
G
Tp53inp2tm1a allele
FRT
loxP
En2 SA
IRES
lacZ
pA
hbactP
neo
X ROSA- Flp
X Th;Cre
H
Relative levels of Tp53inp2 mRNA (normalized to 18S rRNA)
***
LacZ
Cre

Figure S2: *Tp53inp2* mRNA interacts with the TrkA complex to regulate its function. Related to Figure 2. A, RNA immunoprecipitation performed on sympathetic neuron lysates using antibodies for pan-Trk, or normal mouse IgG. Levels of *Tp53inp2* were analysed by RT-qPCR, normalised as fraction of total input and expressed as fold over IgG. Data are presented as average ±s.e.m. multiple t-test (*n*=4, **P<0.01 n.s. = not statistically significant). (See also **Figure 2A**). **B**, RNA immunoprecipitation performed on sympathetic neuron lysates using antibodies for HuD or normal mouse IgG. Levels of *Tp53inp2 or Impa-L* mRNAs were analysed by RT-qPCR, normalised as fraction of total input and expressed as fold of control IgG. Data are presented as average ±s.e.m. multiple t-test (*n*=4, ****P<0.0001). **C,** Western blot analysis of HuD immunoprecipitation in sympathetic neurons. **D**, Western blot analysis of Trk co-immunoprecipitation experiments in PC12 cell lysates. HuD co-immuno-precipitates with Trk. Images separated by vertical lines are taken from the same exposure. Low exposure of HuD in input sample shows presence of duplet (*n*=3). **E,** RNA immunoprecipitation performed on sympathetic neuron lysates using antibodies for NCAM or normal mouse IgG. Levels of *Tp53inp2* were analysed by RT-qPCR and expressed as fold over IgG. Data are presented as average ±s.e.m. Unpaired two tailed *T*-test with Welch's correction (*n*=4, n.s. = not statistically significant). **F**, Western blot analysis of NCAM immunoprecipitation in two representative sympathetic neuron lysates. **G**, Schematic for the generation of the transgenic $Tp53inp2^{fl/fl}$ and $Th\text{-}Cre;Tp53inp2^{fl/fl}$ mice. $Tp53inp2^{tm1a}$ mice were first crossed with the ubiquitously-expressing Flippase 129S4/SvJaeSor-*Gt(ROSA)26Sortm1(FLP1)Dym/J* to excise the LacZ/neo cassette, and then with *Th-Cre* transgenic mice to generate mice lacking *Tp53inp2* in sympathetic neurons ($Th\text{-}Cre;Tp53inp2^{fl/fl}$ mice). Exons are indicated by yellow bars, with UTRs as projecting thin yellow bars. Location of primers used for genotyping indicated as F and R labelled black arrows. **H**, *Tp53inp2* mRNA levels in axons of $Tp53inp2^{fl/fl}$ SCG explants infected with adenovirus expressing LacZ or Cre. Values are normalised by

levels of *18S* rRNA and expressed as percentage of LacZ infected neurons values. Data are presented as average ±s.e.m. one-sample t-test (*n*=3 ***P<0.001). (See also **Figure 2** and **Figure 3**).

Figure S3

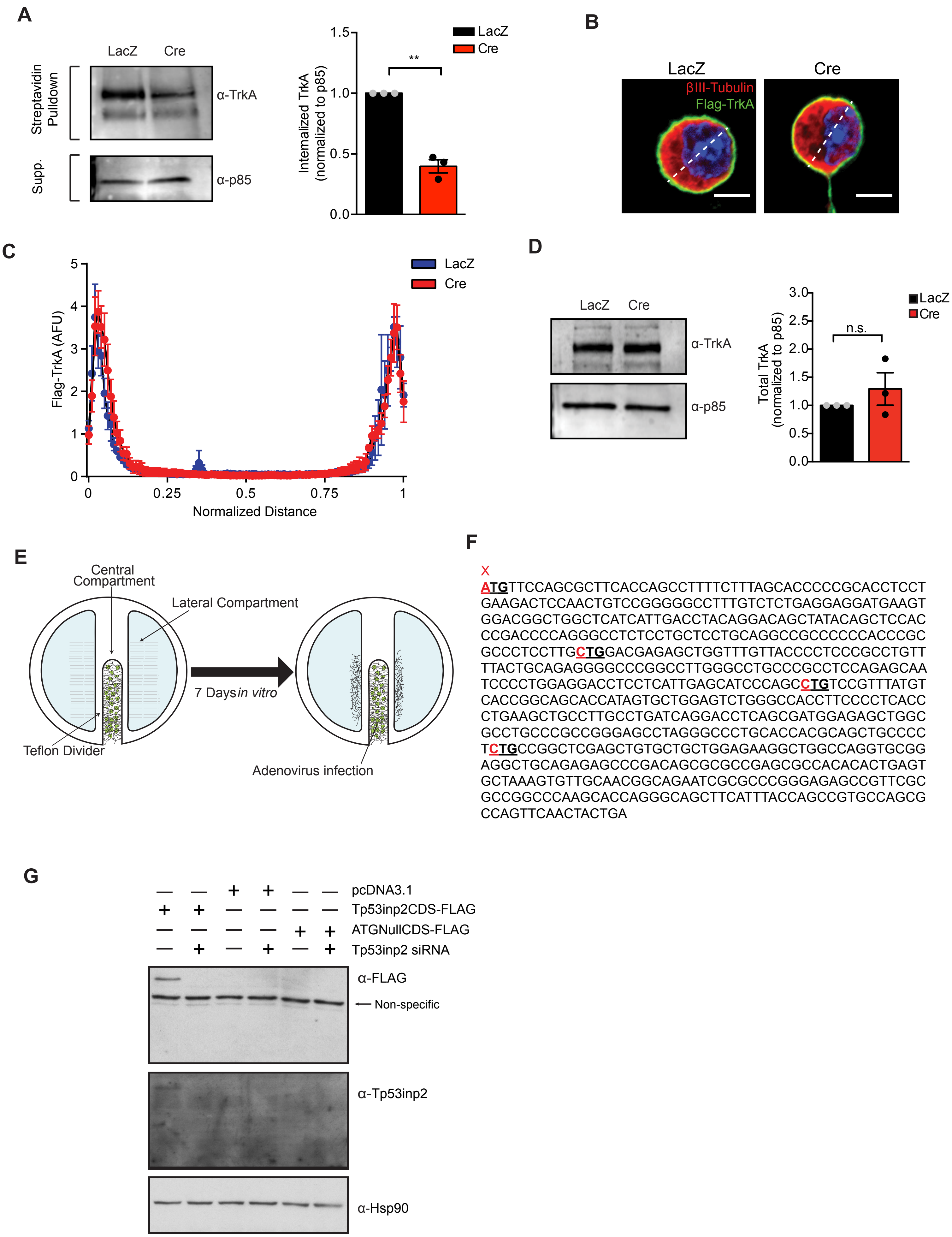
A
LacZ
Cre
Streptavidin Pulldown
Supp.
α-TrkA
α-p85
Internalized TrkA (normalized to p85)
**
1.5
1.0
0.5
0.0
B
LacZ
Cre
βIII-Tubulin
Flag-TrkA
C
Flag-TrkA (AFU)
Normalized Distance
0
0.25
0.5
0.75
1
D
Total TrkA (normalized to p85)
n.s.
E
Central Compartment
Lateral Compartment
Teflon Divider
7 Days in vitro
Adenovirus infection
F
X
ATGTTCCAGCGCTTCACCAGCCTTTTCTTTAGCACCCCCGCACCTCCT
GAAGACTCCAACTGTCCGGGGGCCTTTGTCTCTGAGGAGGATGAAGT
GGACGGCTGGCTCATCATTGACCTACAGGACAGCTATACAGCTCCAC
CCGACCCCAGGGCCTCTCCTGCTCCTGCAGGCCGCCCCCCACCCGC
GCCCTCCTTGCTGGACGAGAGCTGGTTTGTTACCCCTCCCGCCTGTT
TTACTGCAGAGGGGCCCGGCCTTGGGCCTGCCCGCCTCCAGAGCAA
TCCCCTGGAGGACCTCCTCATTGAGCATCCCAGCCTGTCCGTTTATGT
CACCGGCAGCACCATAGTGCTGGAGTCTGGGCCACCTTCCCCTCACC
CTGAAGCTGCCTTGCCTGATCAGGACCTCAGCGATGGAGAGCTGGC
GCCTGCCCGCCGGGAGCCTAGGGCCCTGCACCACGCAGCTGCCCC
TCTGCCGGCTCGAGCTGTGCTGCTGGAGAAGGCTGGCCAGGTGCGG
AGGCTGCAGAGAGCCCGACAGCGCGCCGAGCGCCACACACTGAGT
GCTAAAGTGTTGCAACGGCAGAATCGCGCCCGGGAGAGCCGTTCGC
GCCGGCCCAAGCACCAGGGCAGCTTCATTTACCAGCCGTGCCAGCG
CCAGTTCAACTACTGA
G
pcDNA3.1
Tp53inp2CDS-FLAG
ATGNullCDS-FLAG
Tp53inp2 siRNA
α-FLAG
Non-specific
α-Tp53inp2
α-Hsp90

Figure S3: *Tp53inp2* function in axon growth is translation independent. Related to Figure 2 and 3. A, Membrane proteins in mass cultures of *Tp53inp2*$^{fl/fl}$ neurons, infected with either LacZ or Cre, were subjected to cell surface biotinylation. After NGF stimulation for 30 minutes, surface biotin was stripped off, and internalized TrkA receptors were detected by neutravidin precipitation and TrkA immunoblotting (*Left*). Supernatants were probed for p85 as a loading control. Densitometry analysis (*Right*) of internalized biotinylated TrkA levels, normalised by p85. Values expressed as relative to LacZ-expressing neurons. Data presented as average ± s.e.m., unpaired two-tailed *t*-test (**P<0.01 *n*=3). See also (**Figure 2F, G**). **B**, Representative images of surface Flag-TrkA immunostaining in *Tp53inp2*$^{fl/fl}$ sympathetic neurons infected with adenovirus expressing Cre or LacZ and co-infected with adenovirus expressing Flag-TrkA. Neurons were live-labelled with Flag antibody in the absence of NGF at 4°C. **C**, Surface Flag-TrkA receptor distribution in *Tp53inp2*$^{fl/fl}$ sympathetic neurons infected with adenovirus expressing LacZ or Cre was analysed by measuring the integrated fluorescence values along the longest axis of the cell body using line-plot in ImageJ and normalized to the total cell body fluorescence intensity. Data are presented as averages ± s.e.m. Mann-Whitney two-tailed test (P=0.1089, *n*=3 with at least 15-20 cells analysed per condition per experiment). **D**, Western blot analysis (*Left*) of endogenous TrkA expression in *Tp53inp2*$^{fl/fl}$ neurons infected with LacZ and Cre adenoviruses, with p85 as a loading control. Densitometry analyses (*Right*) of TrkA protein levels normalized to p85. Data presented as average ± s.e.m. Unpaired two-tailed t-test (n.s.= not significant, *n*=3). **E**, Schematic representation of compartmentalised chambers. Neurons are plated in central compartment and axons project into the lateral compartments. For adenoviral infections, adenovirus is supplied to the central compartment after axons start projecting into the lateral compartments. See also (**Figure 2 and 3**). **F**, Nucleotide sequence of ATGNull CDS mutant sequence of *Tp53inp2* rat mRNA. Mutations are indicated by red underlined characters; deletion of adenosine from initial translational start codon and

substitution of adenosine to cytosine in subsequent in-frame ATG codons. See also (**Figure 3C, D**). **G,** Western blotting on lysates of PC12 cells transfected with Tp53inp2CDS-1xFlag, ATGNullCDS-1xFlag or pcDNA3.1 as control. Cells were also transfected with siRNA for *Tp53inp2* mRNA or non-targeting control siRNA (*n*=3). See also (**Figure 3C, D**).

Figure S4

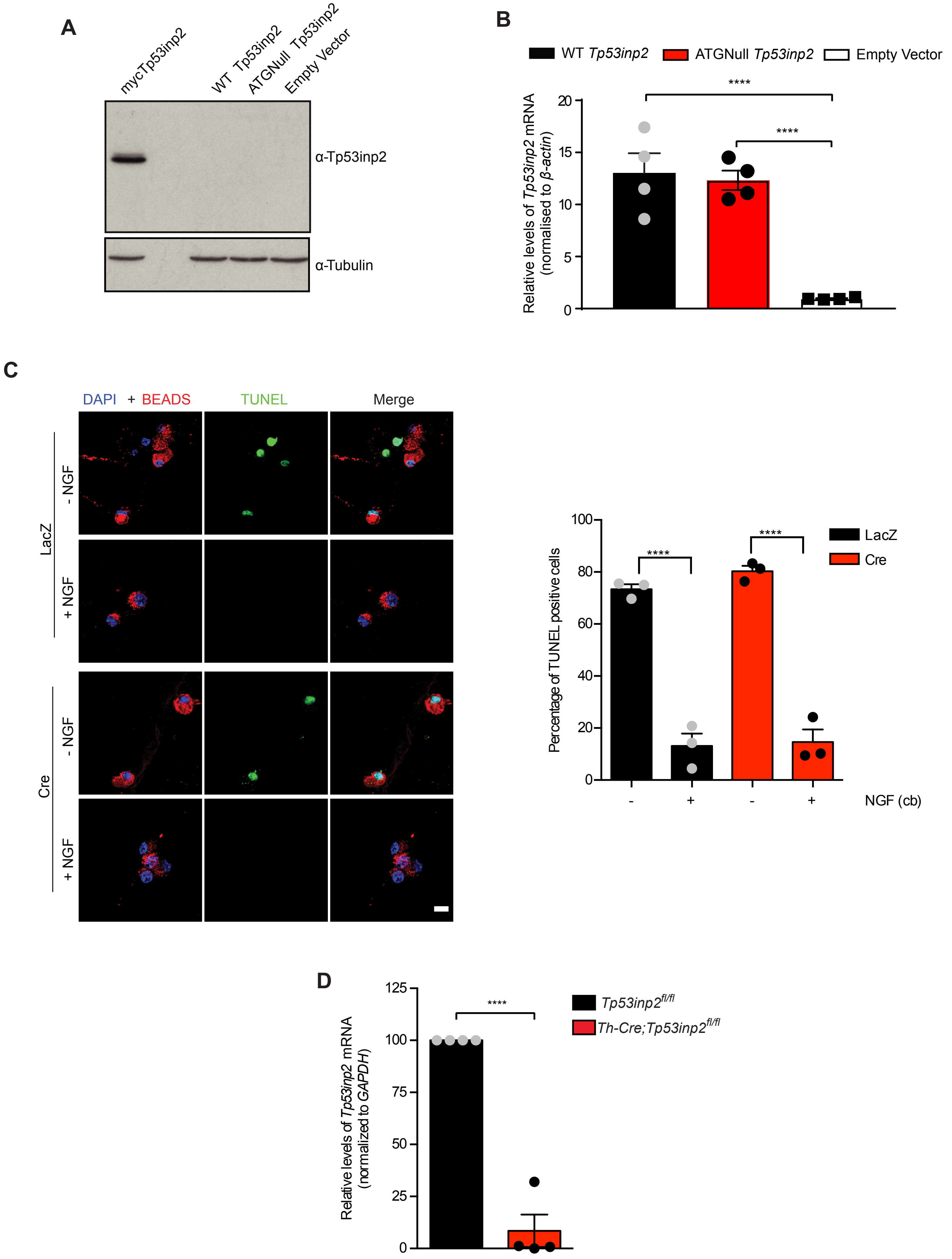

A
mycTp53inp2
WT Tp53inp2
ATGNull Tp53inp2
Empty Vector
α-Tp53inp2
α-Tubulin
B
WT Tp53inp2
ATGNull Tp53inp2
Empty Vector
Relative levels of Tp53inp2 mRNA (normalised to β-actin)
****
****
0
5
10
15
20
C
DAPI + BEADS
TUNEL
Merge
LacZ
Cre
- NGF
+ NGF
Percentage of TUNEL positive cells
LacZ
Cre
****
****
0
20
40
60
80
100
-
+
-
+
NGF (cb)
D
Relative levels of Tp53inp2 mRNA (normalized to GAPDH)
Tp53inp2fl/fl
Th-Cre;Tp53inp2fl/fl
****
0
25
50
75
100
125

Figure S4: *Tp53inp2* is essential for the growth of NGF-responsive sympathetic neurons. Related to Figure 3 and 4. A, Western blotting analysis of PC12 cell lysates transfected with vectors expressing mycTp53inp2CDS, WildType *Tp53inp2* mRNA, full length ATGNull *Tp53inp2* or empty vector. (*n*=3). See also (**Figure 3C, D**). **B**, Expression levels of *Tp53inp2* mRNA in PC12 cells transfected with vectors expressing full length WildType *Tp53inp2* mRNA, full length ATGNull *Tp53inp2* or empty vector, as a control. Levels of *Tp53inp2* mRNA were analysed by RT-qPCR, normalised to levels of *β-actin* mRNA and expressed as fold over control *Tp53inp2* mRNA levels. Data are presented as average ±s.e.m. Ordinary one-way ANOVA (*n*=4,****P<0.0001). (See **Figure 3C, D**). **C,** *(Left)* Cell bodies of compartmentalised cultures of *Tp53inp2*$^{fl/fl}$ neurons infected with adenovirus expressing LacZ or Cre were treated with NGF (25 ng/ml) or anti-NGF antibody for 48 hours. Neurons that had projected to axonal chambers were identified through the uptake of fluorescent beads (red) supplied to the axon chambers. Neuronal apoptosis was detected using TUNEL staining. Scale bar 5 µm. *(Right)* Quantification of neuronal cell death in *Tp53inp2*$^{fl/fl}$ sympathetic neurons expressing Cre or LacZ and NGF supplied to the cell bodies (cb)**.** Data are presented as averages ± s.e.m Ordinary two-way ANOVA Tukey's multiple comparisons, (*n*=3, each data point represents the average of at least 30 cells per condition per experiment, ****P<0.0001). See also (**Figure 3E**). **D**, SCGs were harvested from P0.5 *Th-Cre;Tp53inp2*$^{fl/fl}$ mice and *Tp53inp2*$^{fl/fl}$ littermates. Levels of *Tp53inp2* mRNA were analysed by RT-qPCR, normalised by GAPDH and calculated as percentage of the *Tp53inp2*$^{fl/fl}$ values. Data are presented as average ±s.e.m. unpaired two-tailed t-test *(n*=4 mice per genotype ****P<0.0001). (See also **Figure 4**).

Table S1: List of Tp53inp2 proteotypic peptides identified in Tp53inp2 immuno-precipitate. Related to Figure 1

Peptide sequence	*m/z*	Mass error (ppm)	Charge	Retention time (min)	Ion score (Xcorr)	Main fragment ions (*m/z*)
**ALHHAAAPMoxPAR**	420.2205	-0.3	3	7.3	3.67	343.208 (y3), 530.283 (b5), 601.320 (b6), 672.357 (b7)
**HQGSFIYQPCQR**	507.5734	-0.4	3	25.1	3.58	642.336 (a6), 688.321 (y5), 560.260 (y4), 670.331 (b6), 851.381 (y6)
**ALHHAAAPMPAR**	414.8897	+1.6	3	10.3	4.97	343.208 (y3), 530.283 (b5), 601.320 (b6), 672.357 (b7), 571.302 (y5), 474.249 (y4)

Tp53inp2 tryptic peptides identified by data-dependent nanoLC-MS/MS analysis on a Tp53inp2 immunoprecipitate. Experimental *m/z*, mass error, chromatographic retention time, peptide score according to the Sequest search engine and suitable fragments for pseudo-SRM targeted analysis (based on both ion abundance and specificity) are reported.

Table S2: List of primers used in this study. Related to STAR Methods

Application	Primer Name	Primer Sequences
Genotyping	Tp53inp2LoxP3F	GATCAGGACCTCAGCGATGG
Genotyping	Tp53inp2LoxP3R	GCACCTGGCACAGGTAACTA
Cloning	NotI3UTRTpF	AAT ATA GCG GCC GCG CGC CCC CGC TG
Cloning	XhoI3UTRTp1.2R	TGC TTA CTC GAG CTC TAC CCA GGA GTG TTT ATT CC
Cloning	XhoI3UTRTp2.2R	TGC TTA CTC GAG GCA GGA GAC TGA GTA GTG TTG G
Cloning	XhoI3UTRTp3.1R	CTG GCT CGA GTT AAT GTG CTT TAA ATA GAT TTT TAT TTG
Cloning	NheI5UTRTpF	TAA GCA GCT AGC ACA ATC AGC TGT TGA AGC G
Cloning	BamHI5UTRTpR	TCT TAG GAT CCG GTC AGC GGG TGA AGC
Cloning	BamHINullCDSF	TGA GGA TCC TGT TCC AGC GCT TCA CC
Cloning	NotITpCDSR	TTC GGC GGC CGC TCA GTA GTT GAA CTG GCG
Cloning	KpnIRescueF	ACA GGT ACC ACA ATC AGC TGT TGA AGC
Cloning	EcoRVRescueR	GAC GAT ATC TGT GCT TTA AAT AGA T
Cloning	BamHINullCDSF	TCA GGA TCC TGT TCC AGC GCT TC
Cloning	BamHITpCDSF	TTC AGG ATC CAT GTT CCA GCG CTT C
Cloning	NotITpCDS1xFlagR	AAT GCG GCC GCT CAC TTG TCG TCA TCG TCT TTG TAG TCG TAG TTG AAC TGG CG
Cloning	XbaITpCDS2xFlagR	CGT ATC TAG ATC ACT TAT CGT CGT CAT CCT TGT AAT CCT TAT CGT CGT CAT CCT TGT AAT CGT AGT TGA ACT GGC
Cloning	KpnITpCDSF	GAT CGG TAC CAT ATG TT CCA GCG CTT CAC C
RT-qPCR	qTpF1	GCA CCT CCT GAA GAC TCC AA
RT-qPCR	qTpR1	GGG TAA CAA ACC AGC TCT CG
RT-qPCR	qTpF2	GAA GCT GCC TTG CCT GAT C
RT-qPCR	qTpR2	CCG TTG CAA CAC TTT AGC AC
RT-qPCR	qTpF3	CCT CAT TGA GCA TCC CAG CA
RT-qPCR	qTpR3	CAG CTC TCC ATC GCT GAG G
RT-qPCR	qTpUTRF1	AAC CTT GTT GCT GTC CCA AG
RT-qPCR	qTpUTRR1	GAG CCA AAT GCC CTA TCA AA
RT-qPCR	qTpUTRF2	GGG CTC CTT CTT GCT GCT TA
RT-qPCR	qTpUTRR2	CCT GCT CCC TTC TGC TTC TC
RT-qPCR	qβActF1	ATG GAT GAC GAT ATC GCT GCG
RT-qPCR	qβActR1	GGT GAC AAT GCC GTG TTC AAT
RT-qPCR	qβActF2	TGT CCC TGT ATG CCT CTG GT
RT-qPCR	qβActR2	ATG TCA CGC ACG ATT TCC CT
RT-qPCR	qGFPF1	GAC GTA AAC GGC CAC AAG TT

RT-qPCR	qGFPR1	AAG TCG TGC TGC TTC ATG TG
RT-qPCR	qIVTF1	GTG TTA ACT TCC GAC TCC TCG C
RT-qPCR	qIVTR1	GTG ATG TCA AAC GAC GCA GC
RT-qPCR	qmCherryF1	GAG GGC ACC CAG ACC GCC AA
RT-qPCR	qmCherryR1	ACG CCG CCG TCC TCG AAG TT
RT-qPCR	qMsTpF1	GAG GAG GAT GAA GTG GAC GG
RT-qPCR	qMsTpR1	GGG AGG GGT AAC AAA CCA GC
RT-qPCR	qMsTpF2	GAG GAT GAA GTG GAC GGC TG
RT-qPCR	qMsTpR2	ACC AGC TTC TCG TCC ATC AAG
RT-qPCR	qMsTpF3	GAA GTG GAT GGC TGG CTC AT
RT-qPCR	qMsTpR3	TGC TGG GAT GCT CAA TGA GG
RT-qPCR	qHuTpUTRF1	CTC CCC TTC CTT CTG CCT TG
RT-qPCR	qHuTpUTRR1	TGC AGC TTC TCA ACG TGT CT
RT-qPCR	qHuTpUTRF2	TAG GGC CCC AGT AGT TGA CA
RT-qPCR	qHuTpUTRR2	GGC AGT GAG GAG TAG GCA AG
RT-qPCR	qGAPDHF1	CCT GCA CCA CCA ACT GCT TA
RT-qPCR	qGAPDHR1	CCA CGA TGC CAA AGT TGT CA
RT-qPCR	q18sF1	CGC CGC TAG AGG TGA AAT TC
RT-qPCR	q18sR1	TTG GCA AAT GCT TTC GCT C
